# Genome-wide evolutionary selection pressures acting on *Pseudomonas aeruginosa* residing in different environments

**DOI:** 10.1093/narmme/ugaf040

**Published:** 2025-11-29

**Authors:** Pok-Man Ho, Rahan Rudland Nazeer, Martin Welch

**Affiliations:** Department of Biochemistry, University of Cambridge, Tennis Court Road,Cambridge, CB2 1QW,United Kingdom; Department of Biochemistry, University of Cambridge, Tennis Court Road,Cambridge, CB2 1QW,United Kingdom; Department of Biochemistry, University of Cambridge, Tennis Court Road,Cambridge, CB2 1QW,United Kingdom

## Abstract

*Pseudomonas aeruginosa* is an opportunistic pathogen, commonly associated with the airways of people with cystic fibrosis (CF) and in the wider environment too. In this work, we interrogate the International Pseudomonas Consortium Database (IPCD) to ask the question of whether CF-associated isolates display different patterns of evolutionary selection compared with environmental isolates. We do this by analysing *d_N_*/*d_S_* for each open reading frame (ORF) in the CF-associated and environmental IPCD isolates. Most ORFs displayed a pronounced signature of negative selection (i.e. the ORFs were strongly conserved). However, 373 ORFs displayed non-negative selection, and of these, 206 manifested differential signatures of selection in the CF-derived and environmental isolates. Functional analysis of the ORFs under selection pressure in the CF airways revealed a statistically significant enrichment of enzymes catalysing reactions at metabolic branchpoints. More fine-grained analyses revealed niche-specific selection pressures in individual domains and protein surfaces. Finally, we show that gene loss in the *psl* biosynthetic gene cluster correlates with the presence of loss-of-function mutations in the mucoidy regulator, *mucA*. We speculate that elevated alginate production due to these *mucA* mutations compensates for the loss of Psl production in these isolates.

## Graphical Abstract

**Graphical Abstract:**
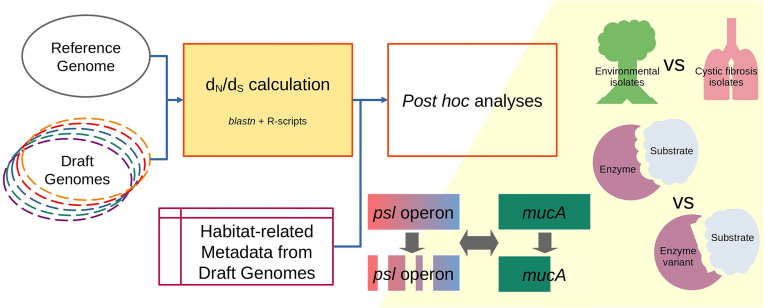

## Introduction

*Pseudomonas aeruginosa* is an opportunistic human pathogen renowned for its ability to adapt to new environments. A good example of this is when environmental strains invade and colonize the airways of people with cystic fibrosis (pwCF) [1, 2]. It is not yet clear why pwCF exhibit such a predilection for infection by *P. aeruginosa*, and there are probably several contributory factors to this. Nevertheless, collectively, a number of studies over the last decade or so have revealed a number of open reading frames (ORFs) encoded by *P. aeruginosa* that appear to encode functions important for CF airway infection.

Commonly encountered loss-of-function mutations in isolates from pwCF has revealed a set of ‘pathoadaptive’ genes that are potentially dispensable for colonization [2–4]. However, this approach tells us little about which genes might be essential for colonization. In 2015, Turner *et al.* began to directly address this using an experimental system that combined Tn-seq and Monte Carlo simulations to identify genes that are essential, conditionally essential and non-essential for growth in different culture media, including artificial sputum medium and expectorated CF sputum (albeit, only from a single patient) [5]. More recently, Weimann *et al.* (2024) employed Bayesian simulations of the pan-genome to identify differential patterns of genomic adaptation in CF and non-CF associated *P. aeruginosa*, enabling the authors to propose a list of genes exhibiting high mutational burdens [2]. However, no previous reports have explicitly investigated whether isolates of *P. aeruginosa* from pwCF and isolates from the environment exhibit differential signatures of selection.

Several approaches have been used to quantitatively estimate the evolutionary selection pressure acting on an ORF, but perhaps the most widely used of these involves calculation of the ratio *d_N_/d_S_* [6, 7]. This is a likelihood-based approach that quantifies the ratio of observed:expected non-synonymous substitutions (*d_N_*) with the ratio of observed:expected synonymous substitutions (*d_S_*) when comparing a sample sequence to a reference sequence. In principle, *d_N_*/*d_S_* values can range from zero to infinity, with a value of 1 representing drift (i.e. no net selection pressure), a value < 1 representing negative selection (also known as stabilizing or purifying selection) and a value > 1 representing directional, disruptive, or positive selection. Although positive selection is often associated with change/gain-of-gene-function, it is important to appreciate that it can also be associated with loss-of-gene-function, provided that the mutation confers a fitness advantage in a particular environment. With suitable datasets in hand, the calculation of *d_N_/d_S_* is not challenging—although for genome-wide analyses, especially those involving large numbers of genomes—this does require considerable computing power.

To date, most *d_N_/d_S_* analyses have relied on simulations with embedded assumptions. For example, Wilson and collaborators used simulation-based *d_N_/d_S_* estimation to study selection pressures in *Mycobacterium tuberculosis* [8, 9]. Their approach assumed that each sample and reference ORF have equivalent length. This analysis necessarily excludes orthologs that contain indels, and does not capture events of complete gene loss, as is frequently observed in pathoadapted strains [10, 11]. Another approach, known as ‘Tajima’s D’ [12, 13], also employs simulations of DNA polymorphisms to estimate the selection pressure. To run this type of simulation, certain evolutionary parameters are assumed such as the average nucleotide substitution rate. However, estimation of such rates is not trivial. For example, we know that *P. aeruginosa* isolates from environments with no associated human activity generally exhibit lower genetic diversity (and therefore, presumably, also lower nucleotide substitution rates) compared with isolates derived from clinical settings [14]. Furthermore, given the relative scarcity of *P. aeruginosa* isolates from pristine environments cf. their abundance in anthropic niches, such conclusions are potentially skewed by sample size issues [15–17].

In the current work, we develop a simulation-free *d_N_/d_S_*-based model to estimate selection pressure acting on a DNA sequence across a discrete moving window. Crucially, our approach is tolerant of indels and gene loss. This allowed us to screen the International Pseudomonas Consortium Database (IPCD) and to map the selection pressures acting on each ORF at single codon resolution. By segregating the *P. aeruginosa* isolates in the IPCD into CF-associated and environmentally derived groupings, we show that certain genes display infection-specific signatures of selection. Many of these genes have not previously been identified as being infection-associated. The high level of granularity achieved by our approach also allows facile mapping of the *d_N_/d_S_* values onto experimentally derived or predicted protein structures, thereby revealing regions of potential functional importance that are not evident through amino acid conservation analyses.

## Materials and methods

Our *d_N_/d_S_* pipeline is shown in Supplementary Fig. S1A. Our approach for *d_N_/d_S_* calculations also allows us to analyse sequences with in-frame indels (Supplementary Fig. S1B). This is because in-frame indels do not necessarily impede protein function [18]. For simplicity, and while we recognize that frameshifts towards the 3′ end of genes do not always lead to loss-of-function, we took a conservative decision to exclude all frame-shifted sequences. Our pipeline is available at https://github.com/ph-u/dNdS_scan and further details are included in Supplementary Information.

Coding sequence (CDS) variations between different environments. Each annotated CDS was matched against our custom database through BLAST. The database consists of the 854 *P. aeruginosa* genomes collected by the IPCD in their initial data release. However, we only included output from 483 genomes which were identified in the accompanying IPCD metadata as being from CF-associated or environmental isolates. Differences between the sequence of each isolate and the respective PAO1 reference genome were annotated and quantified to identify indels. These annotations also enabled insights into gene presence/absence in different habitats.

Evolutionary selectivity (*d_N_/d_S_*) analysis. Applying the equation proposed by Nei and Gojobori [6] to the 5586 indexed ORFs in the PAO1 reference genome, we took a sliding sequence window approach on top of the usual whole [gene] sequence *d_N_/d_S_* calculation. Use of a sliding window enabled residue-by-residue resolution and was informative in quantifying non-uniform signals of habitat-specific selection pressure along each gene.

Contrasting habitat-specific contributions between metabolic pathways and branch points. A list of metabolic reactions associated with enzymes manifesting habitat-specific selection signals was highlighted on the PAO1 KEGG pathway map (pae01100). We identified whether each metabolic reaction on the map was part of a single metabolic pathway, or whether it was associated with a metabolic branchpoint leading into or out of a pathway. A Wilcox rank sum test was carried out to estimate the statistical significance of metabolic reactions enriched in each group.

## Results

### CF-associated and environmental isolates in the IPCD

**Figure 1.**
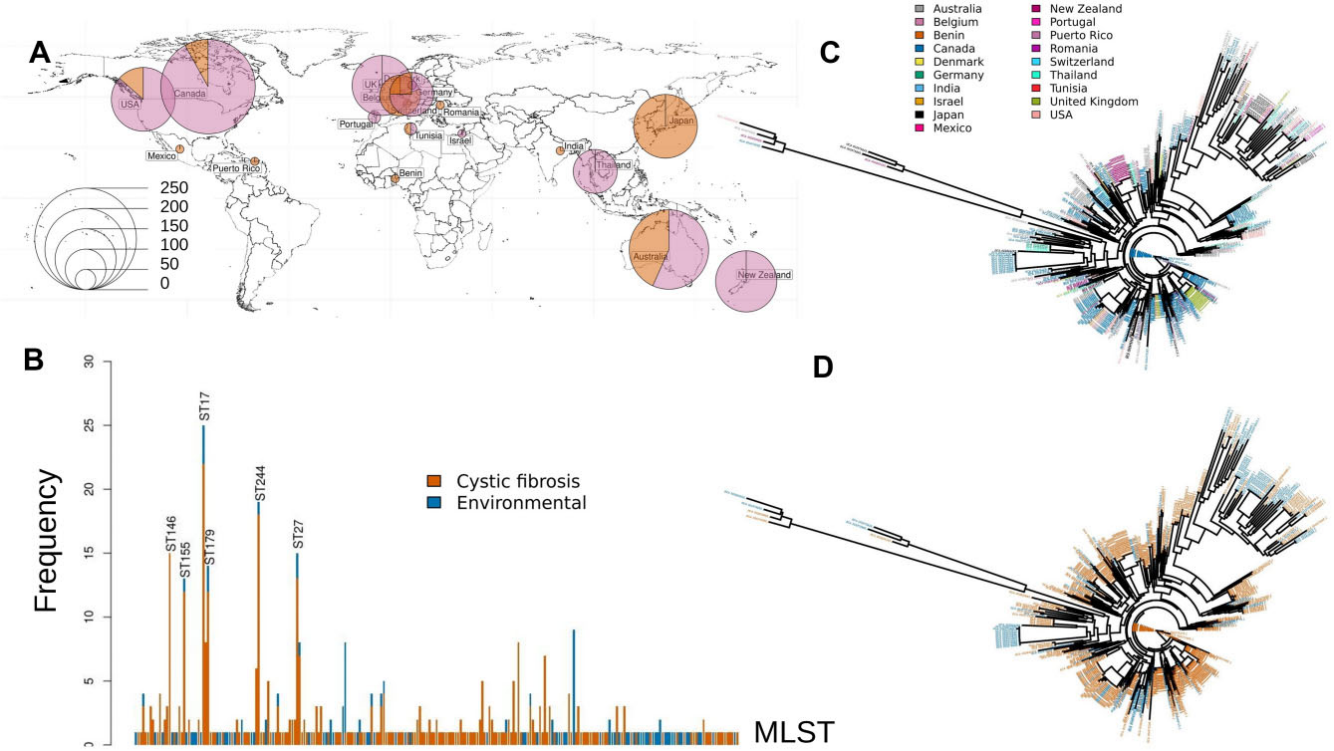
Geographic distribution and phylogenetic relationships of the CF-associated and environmental isolates investigated in this study. (**A**) Geographical distribution of isolates. The size of each pie chart indicates the number of isolates from each location, as shown. Pink; CF-associated isolates, orange; environmental isolates. (**B**) Distribution of MLSTs among the isolates. Note that the majority of isolates had a unique MLST. Colour code: isolates from pwCF (orange); isolates from the environment (blue). (**C**) MLST-based phylogeny of the isolates coloured by geographical source, as indicated. (**D**) MLST-based phylogeny of the isolates coloured according to whether the isolates are sourced from pwCF (orange) or the environment (blue).

Using PAO1 as a reference sequence, we calculated *d_N_/d_S_* for all query genomes annotated in the IPCD as being CF-associated or environmentally derived (Bioproject number: PRJNA325248). The flow scheme summarizing our approach is shown in Supplementary Fig. S1. This [CF + environmental] subset comprised 367 whole genome sequences of CF-associated isolates and 116 whole genome sequences of environmental isolates. The geographic distribution and numbers of isolates from each source are shown in Fig. 1A. We also determined the multi-locus sequence type (MLST) of each isolate (Fig. 1B). This indicated that the majority of isolates had unique MLSTs, and the CF-associated dataset was not dominated by just a few ‘epidemic’ strains. Nonetheless, a small number of sequence types were represented by multiple isolates, although in most cases, these included samples derived from both the environment and pwCF. The MLST data were also used to generate phylogenetic relationships. These indicated that the sequence types showed no particular geographic signatures (Fig. 1C) and that the CF-derived and environmental isolates were polyphyletic (Fig. 1D).

### Calculation of *d_N_/d_S_*

We initially extracted from the PAO1 reference genome sequence all annotated PA loci (Supplementary Fig. S1). We then removed from this collection of genes all untranslated sequences (encoding ribosomal RNA (rRNA), transfer RNA (tRNA), non-coding RNA (ncRNA) etc). This left us with a collection of PAO1-derived ORFs. BLASTN was then used to extract these ORFs from the WGS data of the 367 CF and 116 environmental isolates in the IPCD. Noting that a number of these ORFs also have close paralogues, we took into account the context of each ORF by also examining its flanking regions (100 nucleotides either side of the start and end of the ORF). This allowed us to unambiguously establish the correct context of each ORF. These flanking nucleotides were not taken into account in subsequent *d_N_/d_S_* analyses. *d_N_/d_S_* was then calculated for each whole ORF, and across a rolling window of codons within each ORF, as described in the Supplementary Methods section. Following the BLAST analyses, we also noted that PAO1 encodes 72 ORFs that are absent (or very rare) among the environmental and clinical isolates. These include three phage-derived ORFs, the *wbp* operon and two contiguous uncharacterized operons PA3498-PA3503 and PA3504-PA3514.

### Defining the upper and lower bounds of selection signatures in the dataset

**Figure 2.**
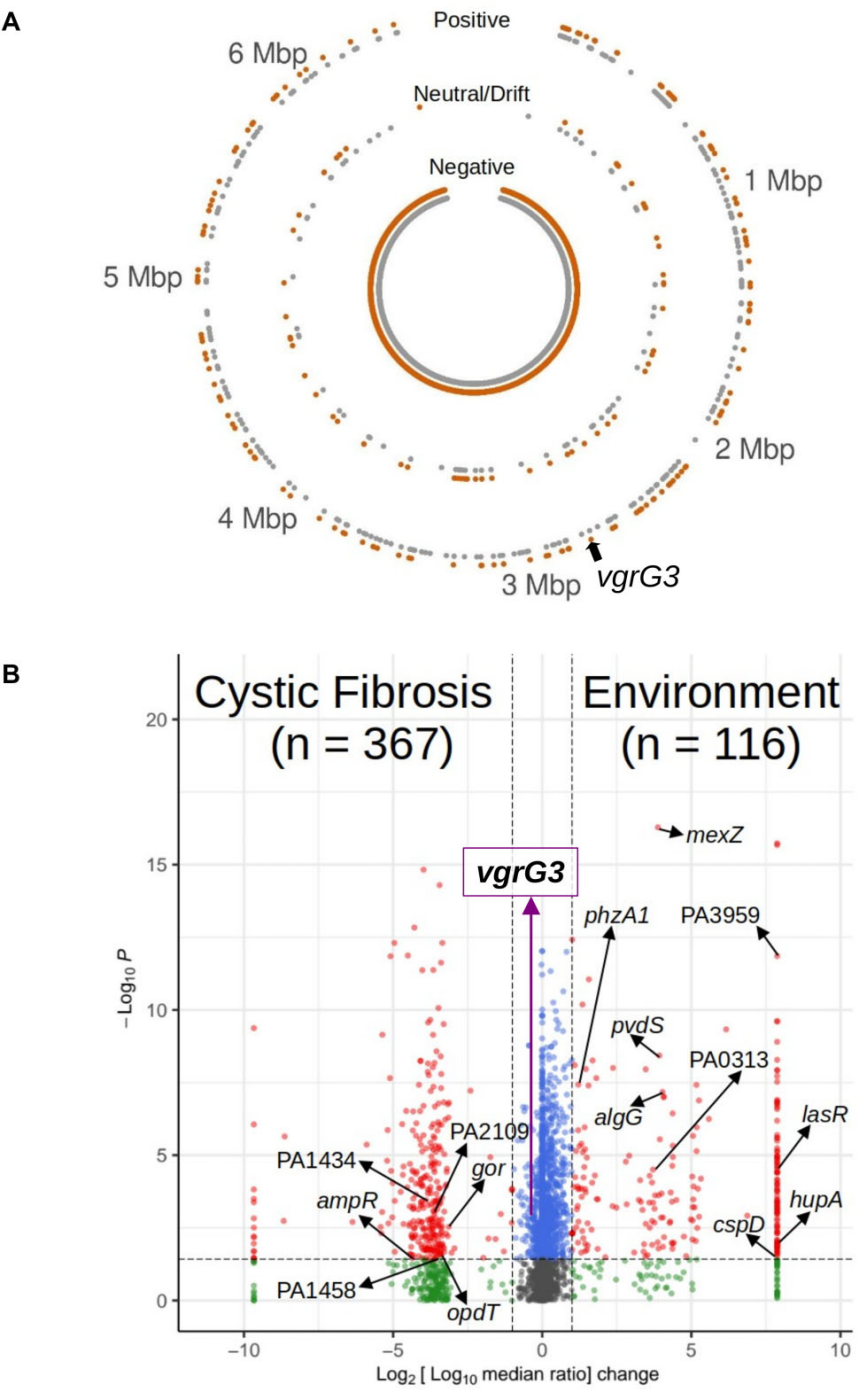
Evolutionary selection pressures acting on ORFs shared by PAO1 and the IPCD query sequences. (**A**) Circular representation of the PAO1 genome with dots showing ORF location and the type of selection [positive (outer rings), neutral (middle rings), and negative (inner rings)] in isolates from pwCF (367 isolates, (grey ●)) and from the environment (116 isolates, (orange ●)). (**B**) Volcano plot comparing ORFs displaying differential selection pressures in isolates derived from pwCF and from the environment. Dots on the right hand side of the plot indicate conservation in environmental isolates (but variability in isolates from pwCF), and dots on the left hand side indicate conservation in isolates from pwCF (but variability in environmental isolates). Source *d_N_/d_S_* data are provided in Supplementary Table S2. ORFs that were previously reported by Weimann *et al.* (2024) as having a high mutational burden in isolates from pwCF are labelled. Note that, in order to visualize the (log_10_ [median *d_N_/d_S_*_(env)_/median *d_N_/d_S_*_(CF)_]) ratios which yielded infinite values in Supplementary Table S2, we arbitrarily set the lower and upper plot boundaries as the maxima of the calculable values ± 1. Key: non-significant and low contrast between CF and environmental isolates (grey ●), non-significant and high contrast between CF and environmental isolates (green ●), significant and low contrast between CF and environmental isolates (light blue ●), significant and high contrast between CF and environmental isolates (red ●). ORFs on the right and left boundaries (i.e. displaying extreme negative selection in one habitat but highly variable selection in the other) are listed in Supplementary Table S2. The *vgrG3* ORF (see body text) is highlighted for easy reference in (A) and (B).

The median *d_N_/d_S_* value was calculated for each whole ORF. Given that the resulting *d_N_/d_S_* values were distributed across a continuum from 0 to >>1, and that very few ORFs manifested a *d_N_/d_S_* value of exactly 1, our first challenge was to define reasonable thresholds for ORFs displaying signatures of negative selection, drift, and positive selection. To do this, we designated the threshold between ‘drift’ and ‘positive selection’ to be the median of all *d_N_/d_S_* values > 1. This yielded an upper threshold of 1.36. However, and given that (as outlined in the next section) the majority of *P. aeruginosa* ORFs had *d_N_/d_S_* values close to zero, the same approach could not be used to define the lower threshold. Instead, we used the reciprocal of the upper boundary (0.73) to delineate the threshold between drift and negative selection. Using these cutoffs, we were able to categorize which ORFs in the CF-associated and environmental isolates display differential selection signatures (Fig. 2A).

### ORFs showing differential *d_N_/d_S_* signatures in CF-derived and environmental isolates

Perhaps not surprisingly, we found that when comparing the ORFs from CF-associated and environmental isolates, most (ca. 96%) of the ORFs had a signature of negative selection i.e. these ORFs had *d_N_/d_S_* values < 0.73. However, 373 ORFs displayed signatures of non-negative selection, and of these, 206 manifested differential signatures of selection in CF-derived and environmental isolates (Supplementary Table S1). Note that for 35 ORFs, *d_N_/*d_S_ could not be calculated. The reasons for this are given in the legend for Supplementary Table S1. A list of the 373 ORFs displaying non-negative selection signatures, along with their respective *d_N_/d_S_* values, is shown in Supplementary Table S2, and the data are visually represented in Fig. 2A.

Of particular interest were those ORFs that displayed contrasting signatures of selection in the CF-associated isolates compared with the environmental isolates. To visualize these, we generated a ‘volcano plot’ by calculating the ratio of the median *d_N_/d_S_* values between the CF and environmental isolates for each ORF (Fig. 2B). As this approach takes no account of the drift boundaries outlined above, Fig. 2B contains 5586 data points (i.e. the total number of ORFs analysed). Interestingly, and among those ORFs displaying conservation (*d_N_/d_S_* < 0.73) in the environmental isolates but variability (*d_N_/d_S_* values > 1.36) in the CF isolates (301 ORFs), and conversely, those displaying conservation in the CF isolates but variability in the environmental isolates (229 ORFs), only 15 were previously reported as having a high mutational burden in pwCF by Weimann *et al.* (2024). A list of the ORFs displaying extreme signatures of differential selection pressures (i.e. those on the far right and far left boundaries of Fig. 2B, respectively) are listed in Supplementary Table S3. Interestingly, of the ORFs that were extremely conserved in CF isolates but variable in environmental isolates, none were previously demonstrated to be essential for growth in artificial sputum medium *in vitro* [5]).

**Figure 3.**
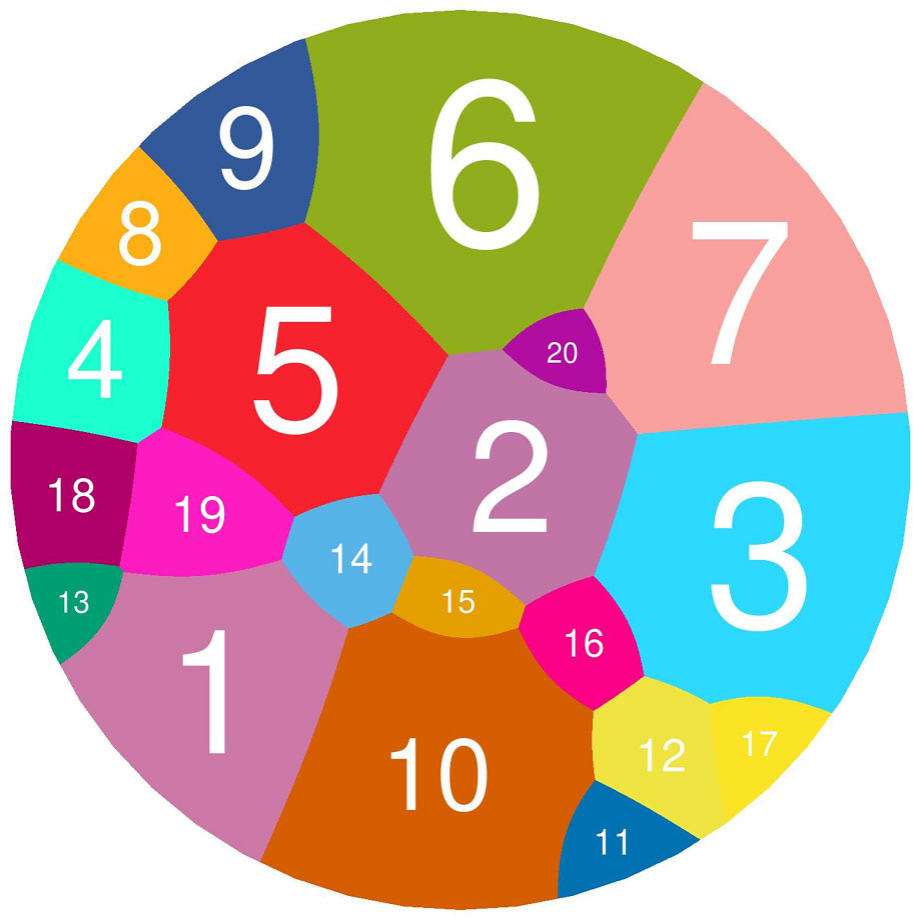
Voronoi map representing the consensus STRING network associated with differential selection in CF and environmental isolates. ORFs represented by the 530 'significant and high contrast' data points in the Volcano plot (Fig. 2B) were subjected to iterative *k*-means clustering as outlined in the body text to yield 20 statistically robust functional clusters (indicated as different colours in the figure). The corresponding STRING map is shown in Supplementary Fig. S2, and the ORFs in each coloured/numbered cluster are shown in Supplementary Table S4. The largest represented functional categories are 1 (no significant functional enrichment), 2 (chemotaxis and signal transduction), 3 (mixed metabolism), 5 (Type III secretion [T3S], virulence, glutathione metabolism), 6 (Type VI secretion [T6S] and mixed metabolism), and 10 (catabolism).

In Fig. 2B, the 530 red data points represent ORFs displaying significant (*P* < 0.1) but contrasting *d_N_/d_S_* values in the environmental and CF-derived isolates. To establish whether there are any trends or patterns (e.g. functional enrichment of ORFs) evident in these data, we carried out a STRING analysis, which yielded a network of interactions between the ORFs. To optimize this functional categorization, we used a *k*-means clustering approach. In essence, the AI-based STRING algorithm was instructed to designate 20 putative functional clusters. Next, the algorithm was asked to generate a much larger number (e.g. ca. 70) of functional clusters from the initial 20 obtained in the previous step. The output of this step was then used as an input to regenerate another 20 functional clusters, which were saved. This process was then iterated a further six times (for *N* = 7 iterations in all). The logic here was that, being AI-based, the output of each iteration is not strictly deterministic, and so this approach yields a more statistically reliable network of functional clusters. The consensus network is shown in Supplementary Fig. S2 and is represented as a Voronoi map in Fig. 3. A list of the ORFs in each consensus cluster is shown in Supplementary Table S4.

**Figure 4.**
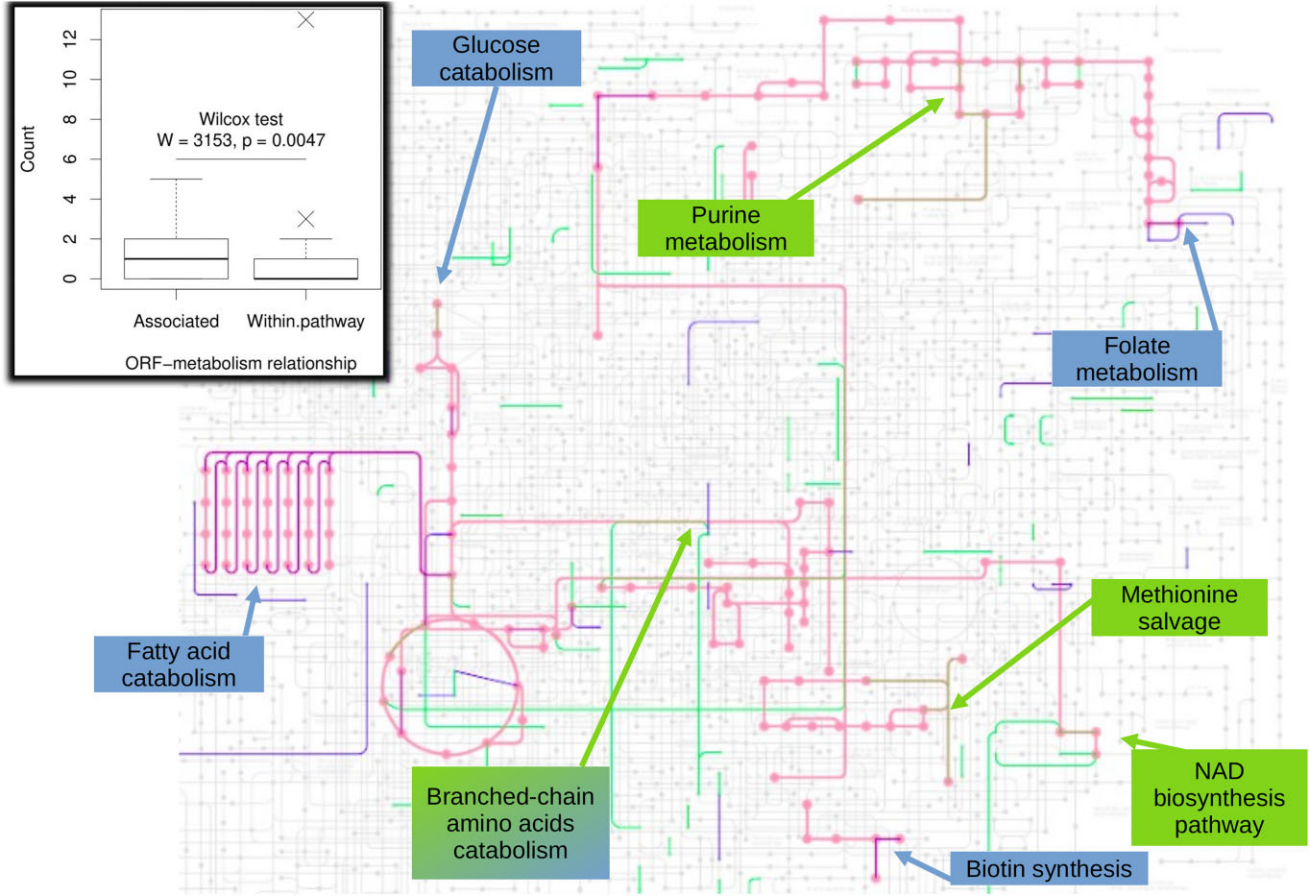
KEGG map highlighting the metabolic pathways displaying habitat-specific selection pressure in CF-derived and environmental isolates from the IPCD. The figure shows the metabolic pathways influenced by habitat-specific selection pressures (pink) mapped onto the comprehensive metabolic pathways map hosted by the KEGG [19]. Reactions associated with CF-derived isolates are shown in green whereas reactions associated with environmental isolates are shown in blue. Note that some reactions are overlain in more than one colour because they are differentially selected in CF- and environmental isolates, leading to different shades of colouration, such as purple and brown. Selected domains of metabolism associated with key signatures of selection are boxed (in green or blue, as appropriate) and identified. The full list of metabolic pathways (and their associated ORFs) that are differentially affected in the CF- and environmentally derived isolates is shown in Supplementary Table S5. *Inset*. Boxplot showing the distribution of ORFs catalyzing either main-sequence pathway reactions (‘within pathway reactions’) or branchpoint reactions (‘associated reactions’). Note that the furthest outlier in this plot are the reaction(s) catalyzing β-oxidation of fatty acids (KEGG code M00087). The enzymes catalyzing these reactions are highly conserved in environmental isolates, but are subject to positive selection in CF-associated isolates. The other outlier was associated jointly with enzymes of the methylcitrate cycle (M00982) [20, 21] and enzymes of adenine nucleotide degradation (M00958).

Next, and to extract meaningful trends from the STRING analyses (e.g. the biological pathways affected) we mapped the ORFs in the individual STRING clusters onto the KEGG pathways framework (pae01100). This revealed that distinct pathways are under differential selection in the CF-derived and environmental isolates (Fig. 4). Interestingly, we noted that habitat-specific selection pressures appeared to be primarily associated with reactions at metabolic branchpoints. To further test this observation, we designated each ORF as being either embedded in a single metabolic pathway or associated with a metabolic branchpoint leading into or out of a pathway (Supplementary Table S5). Analyses of these data using a rank-sum test (the Wilcox test) revealed a statistically significant robust enrichment of ORFs associated with metabolic branchpoints (*P* = 0.0047) (Fig. 4, inset).

### **Residue-level analysis of *d_N_/d_S_* reveals a more fine-grained distribution of selection pressure acting on** individual **ORFs**

**Figure 5.**
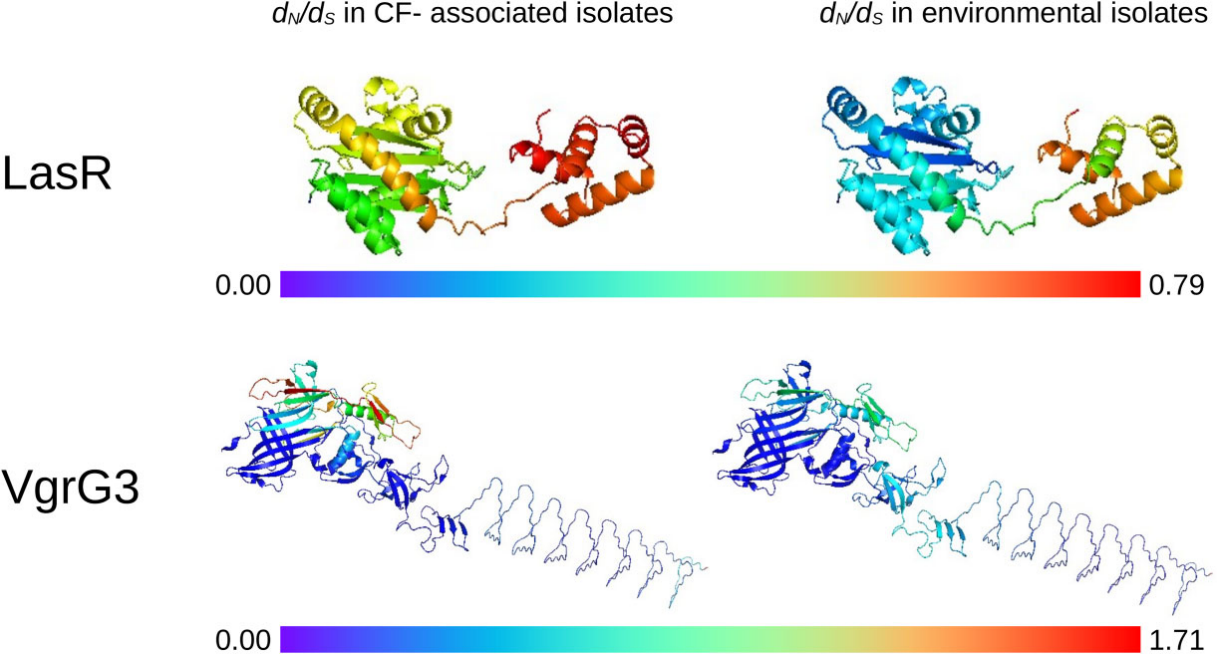
Residue-level *d_N_/d_S_* analysis of two exemplar proteins present in the CF-associated and environmental isolates. The figure shows the *d_N_/d_S_* values, determined at single codon resolution, mapped onto the three-dimensional structures of LasR and VgrG3. Note that although the median *d_N_/d_S_* values of *lasR* and *vgrG3* are infinite and zero (in CF isolates, respectively) and 0.22 and 3.67 in environmental isolates (respectively), from a sliding window perspective, the value for non-synonymous mutations is capped at ‘the maximum finite *d_N_/d_S_* value of + 2’. This is why the maximal *d_N_/d_S_* scale for *vgrG3* is 1.71 in the figure.

In addition to examining the median *d_N_/d_S_* values for ORFs, we also measured *d_N_/d_S_*, residue-by-residue, for each ORF. This resource is now publicly available via Zenodo (https://doi.org/10.5281/zenodo.16393514). This allowed us to map selection pressures onto the (experimental or predicted) three-dimensional structures of the encoded proteins, enabling a better understanding of which domains, surfaces, and residues are under selective pressure in different habitats. As a case study, we present here the high-resolution *d_N_/d_S_* profile of the quorum sensing ‘master regulator’ encoded by *lasR*, and of the Type VI Secretion Machinery protein, encoded by *vgrG3*. These ORFs were chosen because the median *d_N_/d_S_* value for *lasR* indicates that the gene is under strongly negative selection (i.e. is conserved) in environmental isolates (median *d_N_/d_S_* = 0) but exhibits much weaker signature of conservation in CF isolates (median *d_N_/d_S_* = infinite), whereas *vgrG3* exemplifies the opposite trend, being overall negatively selected in CF isolates (median *d_N_/d_S_* = 0.22), but evolving away from the ancestral state in environmental isolates (median *d_N_/d_S_* = 3.67). For each ORF, we mapped the residue-by-residue *d_N_/d_S_* signatures onto the structure of the encoded protein, as predicted by AlphaFold (Fig. 5). The data show several things. First, although the notion of a ‘median *d_N_/d_S_* value’ for an ORF does have some utility, fine-grained analyses indicate that different parts of the encoded protein can be subject to different selection pressures. This is very clear in the case of LasR, where the N- and C-terminal domains of the protein manifest obviously differential selection (Fig. 5). Second, these selection pressures can be different in isolates from different environments. In the case of LasR, for example, the N-terminal OdDHL-binding domain is more strongly conserved in environmental isolates than it is in the CF-associated isolates. The pattern of selection in VgrG3 is even more complex with a more patchy distribution of selection in isolates from the different habitats and localized ‘hotspots’ of variation in the CF-derived isolates. It is possible that this extremely localized selection pattern may reflect the greater diversity of T6SS effectors that are handled by VgrG3 in CF isolates [22].

### A ‘contingency region’ of lost genes in CF isolates

**Figure 6.**
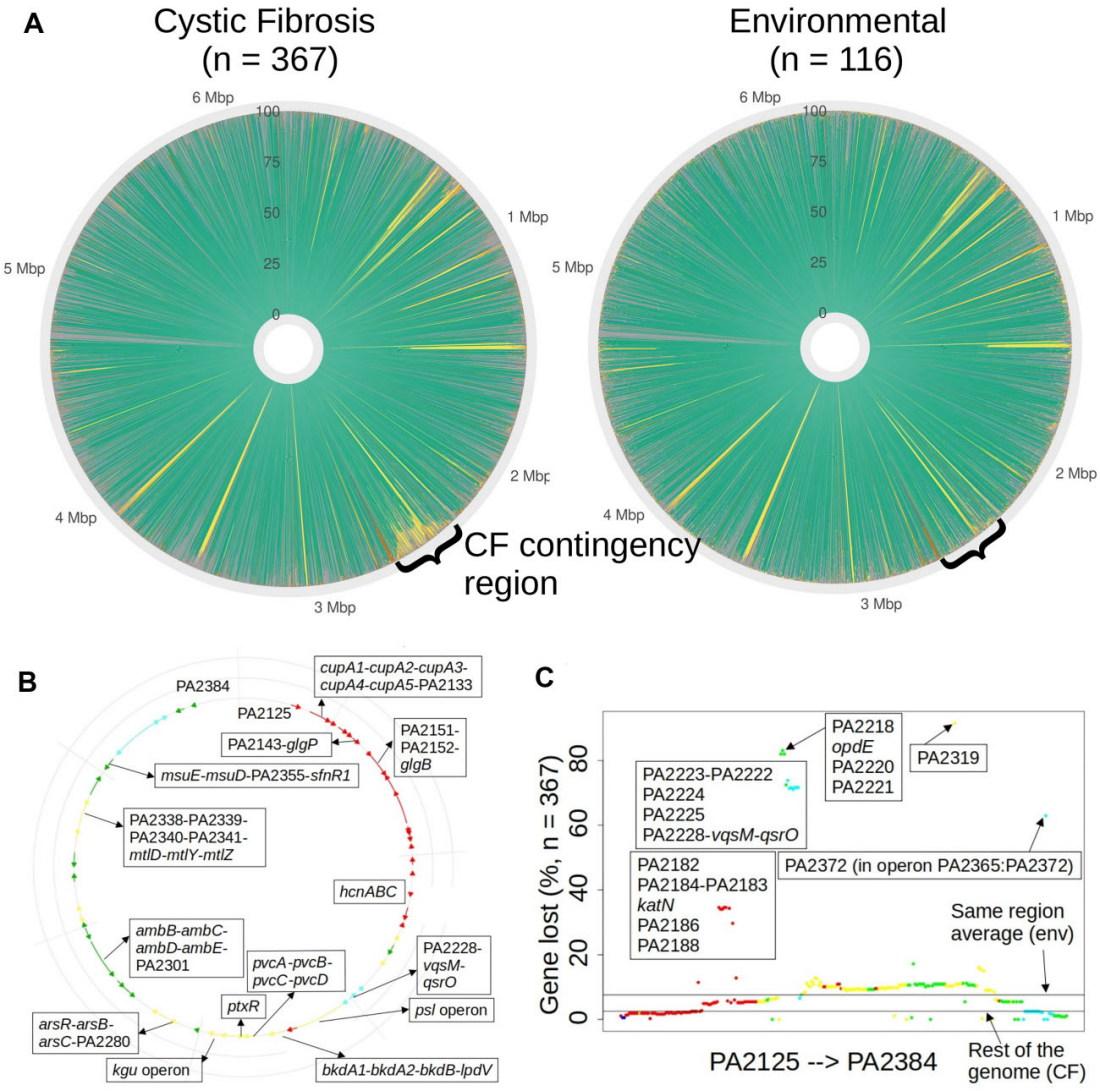
A contingency region prone to loss in CF-associated isolates.(**A**) Circular bar charts showing gene presence/absence in CF- and environmental isolates. Genes were compared based on the genomic arrangement in PAO1. The depth of radially displayed colour indicates the proportion (%) of isolates associated with the following genetic signatures: identical (grey), non-frameshifting indel present but otherwise identical to PAO1 (pink), frameshifting indel present (brown), non-frameshifting indel present and at least one SNP (blue), ORF present in PAO1 genome but absent in subject genomes (yellow), and at least one SNP present (green). (**B**) Visual representation of characterized operons present in the contingency region (PA2125-PA2384). The colour key is linked with the STRING/k-means analyses (https://version-12-0.string-db.org/cgi/network?networkId=bPujOzfKx9Ba): cluster 1 (red), cluster 2 (yellow), cluster 3 (green), cluster 4 (cyan), and cluster 5 (blue). (**C**) Percentage of gene loss of each gene in the contingency region among clinical isolates from pwCF. The colour key follows that in (B) reflecting the designated functional cluster of each gene.

During the early part of these analyses (and prior to the *d_N_/d_S_* calculations) we also noted that some CF-associated isolates exhibited a pronounced pattern of gene loss in one particular ‘contingency region’ [corresponding to the PAO1 loci PA2125-PA2384 (Fig. 6A)]. Gene loss across this region was sporadic but significantly higher than it was in other parts of the genome (Wilcox W = 1 356 116, adjusted *P* << 0.01). Similarly, gene loss in the region was also significantly higher than it was in environmental isolates (Wilcox W = 53 730, adjusted *P* << 0.01). PA2125-PA2384 contains 14 operons, comprising 260 ORFs (Fig. 6B). One particularly common ‘missing operon’ was PA2228-*vqsM*-*qsrO*, which was missing in >70% of isolates from pwCF (Fig. 6C). This operon encodes ORFs associated with quorum sensing and virulence.

To understand the probable physiological role (s) of the ORFs in this contingency region, we carried out a functional clustering using STRING/*k*-means and five designated clusters (indicated as different colours in Fig. 6B and C). This analysis revealed that ORFs involved in Psl exopolysaccharide biosynthesis, biofilm regulation, and Type VI Secretion are more prone to be discarded among (presumably pathoadapted) *P. aeruginosa* strains in the airways of pwCF.

**Figure 7.**
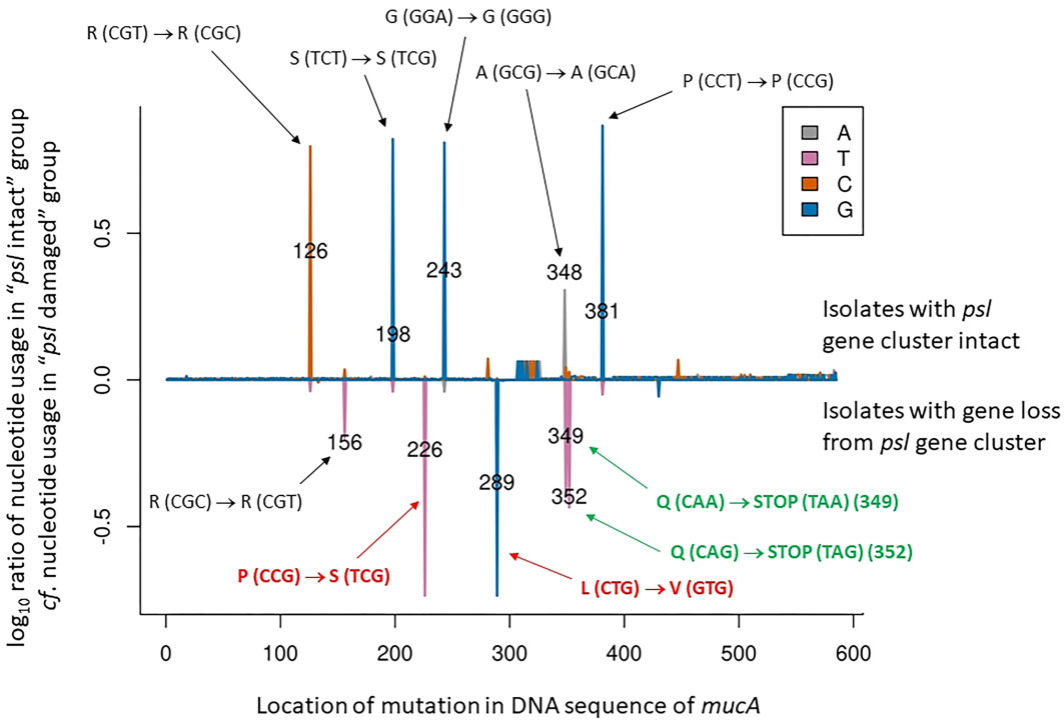
Diminished Psl biosynthetic potential due to *psl* gene loss in the contingency region is accompanied by an increased rate of mutation in *mucA*. The graph shows the locations of mutations in the *mucA* ORF that are enriched in the indicated isolates. Positive values indicate enrichments among the 310 strains that had an intact *psl* gene cluster whereas negative values indicate enrichments among the 57 strains with at least one *psl* gene missing from the *psl* gene biosynthetic cluster. Synonymous mutations are labelled in black, missense mutations in bold red, and nonsense mutations in bold green. Codon annotations refer to the PAO1 *mucA* ORF sequence.

The loss of *psl* biosynthetic genes from the contingency region was intriguing, especially given that Psl is a major contributor to biofilm formation; a phenotype that has been long-associated with the establishment of chronic infections [23–25]. This made us wonder whether the presumed absence of Psl in these strains might be offset by synthesis of another biofilm-associated polysaccharide. One such polysaccharide could be alginate, which is over-produced in many CF isolates as a result of loss-of-function mutations in the anti-sigma factor encoded by *mucA*. We therefore predicted that the strains associated with *psl* biosynthetic gene loss in the contingency region might have a higher frequency of mutations in *mucA*. This was indeed the case (Fig. 7). Although the *mucA* ORF in CF-associated isolates in which the *psl* gene cluster was intact did acquire mutations, the most frequent of these led to synonymous changes. By contrast, in the isolates where *psl* gene cluster integrity was affected, *mucA* acquired missense or nonsense mutations. It seems likely that the latter lead to over-production of alginate, possibly compensating for the absence of Psl in those strains.

## Discussion

In this study, we developed a simulation-free *d_N_/d_S_*-based model to estimate the selection pressure—at single codon resolution and at a whole gene level—acting on each ORF shared between the *P. aeruginosa* type strain (PAO1) and each of 367 CF-associated isolates or 116 environmental isolates of the species. Care was taken to ensure that the geographic distribution of isolates examined was broad, and that the isolates were polyphyletic. By monitoring selection signatures (as opposed to mutation *per se*), our data highlight genes that are subject to differential selection pressures in different habitats (in this case, the CF airways and the wider environment).

Our approach (which is agnostic to evolutionary assumptions) tolerates the presence of non-frameshifting indels and also takes into account the context of each ORF, thereby ensuring robustness in the assignment of ORFs to specific PA loci. This was important because of the presence of paralogs. For example, PAO1 encodes three copies of the type VI secretion lipase immunity protein, designated *tli5b1, tli5b2*, and *tli5b3*. These ORFs share substantial amino acid sequence identity and are encoded adjacent to one another in the genome. At a whole ORF level, *tli5b1* (PA5086), *tli5b2* (PA5087), and *tli5b3* (PA5088) were all conserved in both CF-derived and environmental isolates. The presence of multiple type VI secretion system-related immunity genes *per se* is consistent with the multi-copy ‘stockpiling hypothesis’ [26, 27]. However, patterns of selection across the individual *tli5b* ORFs are highly variable (Supplementary Fig. S3), which emphasises the importance of residue-by-residue *d_N_/d_S_* analyses when interpreting the data.

Perhaps not surprisingly, our data confirm that most ORFs in the PAO1 genome are highly conserved (i.e. are under extreme negative selection) indicative of a trans-niche core genome. Indeed, only 4% of all PAO1-encoded ORFs manifested signatures of habitat-specific differential selection pressure. Most of these ORFs are currently uncharacterized. Interestingly, the majority of genes previously reported to be under ‘high mutational burden’ in isolates from people with CF often displayed only marginal differences in *d_N_/d_S_* compared with the same genes in environmental isolates. For example, Weimann *et al.* proposed a set of 224 ‘pathoadaptive’ genes with high mutational burden based on a Bayesian simulation framework of the *P. aeruginosa* pangenome. However, in our analyses, just 15 ORFs from this list displayed differential habitat-specific selection, and even then, only 9 displayed CF-associated positive selection signatures. This shortlist included known pleiotropic virulence regulators such as *lasR, pvdS*, and *algG*, as well as the AMR determinant *mexZ* [28–34].

Although the focus of this work has primarily been on ORFs that might be labelled ‘pathoadaptive’, the work also sheds light on ORFs which could be ‘enviro-adaptive’ too. Functional clustering using STRING of the putative ‘enviro-adaptive’ ORFs (i.e. those represented as red dots on the right hand side of the volcano plot in Fig. 2B) revealed that the 233 ORFs that were more conserved in environmental isolates (and more variable in the CF-associated isolates) were enriched in functions associated with T2S [e.g. ORFs PA0679, PA3095 (*xcpZ*), and PA3104 (*xcpP*)] and nucleotide manipulation mechanisms (especially ORFs encoding XRE-family DNA-binding and cupin signal-sensing domains). In PA, there are at least eight transcriptional regulators pairing the XRE domain with a C-terminal cupin-sensing domain, many of which repress expression of their neighbouring genes [35]. One such ORF, PA4499 (PsdR), was recently described as also a quorum-sensing regulator [36]. Another pair of putatively enviro-adaptive genes (PA5403 and PA5406) have been implicated in regulating the growth of *P. aeruginosa* under carbon starvation and high-density conditions [37]. By contrast, among the 297 putatively ‘pathoadaptive’ ORFs, i.e. ORFs that were differentially more conserved in CF-associated isolates (and more variable in environmental isolates) we noted enrichments in ORFs linked with [phosphor]sugar/lipid metabolism, chemotaxis, two-component systems, and quorum sensing. One unexpected enrichment was also ORFs encoding a BON domain (e.g. PA2562, PA5182, and PA5183). ‘BON’ is derived from ‘bacterial OsmY and nodulation’, and BON domains are thought to interact with phospholipid membranes [38], aiding in protein localization [39] and resistance towards antimicrobials and heavy-metals [40]. BON domain-containing proteins are not limited to pathogens or specific habitats, suggesting a potential intrinsic adaptation strategy that is common across many prokaryotes [41].

*LasR* is a known ‘hotspot’ for mutation in CF isolates [42, 43]. The reasons for this are not entirely clear but have been variously attributed to the appearance of ‘evolutionary cheats’, or more prosaically, that possession of a functional *lasR* (and its attendant virulence phenotype) may be disadvantageous in the CF setting. Although the appearance of cheats should be positively selected in the short-term, in the longer-term, cheating is expected to display negative frequency-dependent selection. By contrast, if possession of a functional *lasR* is detrimental in the CF airways, loss-of-function *lasR* mutants should be positively selected. Our analysis indicates that *lasR* mutations are indeed positively selected in the CF isolates, favouring the latter (loss-of-virulence) hypothesis. However, the situation is likely more complex than this, especially given recent findings suggesting that *lasR* mutants can enhance CF airway disease progression by promoting inflammation [41].

Other examples of positive selection signatures that may be linked with loss-of-gene-function in CF isolates (Supplementary Fig. S3) include ORFs associated with e.g. Type III Secretion (*pscA1, pscH, pcr4*, and *pscG*). However, the majority of positively selected ORFs in the CF isolate collection (Supplementary Table S3), are either poorly characterized or uncharacterized, so we cannot comment further on the likely functional consequence(s) of the mutations. Instead, we note that the most highly positively selected ORFs in CF isolates were *panB* (encoding an enzyme involved in pantothenate biosynthesis [42, 43], an enzyme (PA2087) probably involved in metabolism of an aromatic compound, and a probable murein transglycosylase (PA3959). Other loci of note in this category include the biofilm regulator, *bfiR* [44] the paerucumarin biosynthetic enzymes *pvcB* and *pvcD* [45] and the key glyoxylate shunt enzyme, *iso*citrate lyase (*aceA*) [46, 47]. These, and other enzymes on the list of 99 positively selected ORFs may play an important role in the CF niche and are deserving of further attention.

Flux through β-oxidation is known to play an important role in adaptation to the human tissue environment, which is rich in fatty acids [48–51]. The resulting acetyl-CoA is then used, partially for energy production (via the TCA cycle) and partially for gluconeogenesis (via the glyoxylate shunt). In this regard, the identification of *aceA* as a subject of CF-associated positive selection was particularly gratifying because the encoded enzyme plays a key role in regulating flux partitioning between the glyoxylate shunt and the TCA cycle. Indeed, and more broadly, we noted a statistically robust signal of habitat-specific selection pressure associated with the enzymes at metabolic branchpoints. This is a gratifying observation, since the importance of branchpoint enzymes in pathway flux control—especially during adaptation to a new nutritional environment—is predicted from metabolic control theory [52].

In the course of this analysis, we also identified a region (encompassing loci PA2125-PA2384 in PAO1) characterized by higher-than-expected gene flux in the CF-associated isolates. The loss of genes from this region is consistent with a signature of ‘pathoadaptation’ [53, 54]. A broader range of genes that partially overlap with this contingency region was also reported in some non-CF clinical isolates (e.g. [55] and [56]), which may confer phage and meropenem resistance [57, 58]. A major hotspot for gene loss in the region was in the *psl* operon. This was unexpected, given the known association of CF strains with the biofilm lifestyle, and the fact that Psl is a major component of biofilm exopolysaccharide [59, 60]. However, we also found that this loss of Psl biosynthetic potential was offset by a higher frequency of mutation in *mucA*, presumably leading to increased production of an alternative, CF-pathognomic polysaccharide, alginate. We cannot comment on whether loss of *mucA* function pre-disposes the cell to *psl* gene degradation, or whether *psl* gene loss precedes *mucA* mutation (thereby compensating for the lack of Psl). If the latter, this raises the question of why the cell might want to down-regulate Psl production? One possibility is that Psl is known to be the more immunostimulatory of the three exopolysaccharides produced by PA [61] so it is tempting to speculate that loss of the Psl biosynthetic genes, as well as other key regulators of virulence, might contribute towards pathoadaptative ‘cloaking’ against the immune system. An obvious corollary is that targeting gene products associated with this region (i.e. genes that are lost at a relatively high frequency) may not prove to be a particularly useful therapeutic strategy in CF [62, 63].

While our analysis has identified ORFs under selection in the CF airway and in the environment, a key future challenge will be in understanding *why* these ORFs are under selection. This is not a straightforward issue to address experimentally, since selection pressures are likely to be diverse and non-uniform across the different isolates examined in this study. For example, these selection pressures may include some or all of the following factors: (i) the change in chemical environment when a ‘naïve’ environmental isolate enters the CF airways, (ii) the nature of the environment that the invading strain is entering from, (iii) the presence of competing microbial species (and the CF airways frequently harbour a diversity of microbes, including bacterial, viral, and fungal species, and indeed, genetic variants of those species, (iv) the presence of host immune systems, (v) antibiotic treatments, (vi) the genetics of the host (GWAS data indicate that some hosts are pre-disposed towards infection by e.g. mucoid strains etc (reviewed in [64]), (vii) the host diet (re: the increasingly recognized ‘gut–lung axis’ [65]), (viii) non-antimicrobial medications such as inhaled DNase and pancreatic supplements (which can also have a profound impact on the lung microbiome [66]), and (ix) the disease status of the individual at the time of sampling (early/late stage, stable/exacerbating, etc). Accessing the role (s) played by each of these selection pressures in shaping the evolutionary trajectory of *P. aeruginosa* will be challenging. However, and encouragingly, we do note that previous work has shown that growth of PAO1 (a ‘domesticated’ strain which has been passaged for decades in ‘standard laboratory media’ such as LB) in artificial sputum medium is accompanied by the accumulation of SNPs that betray a clear selection signature in *d_N_/d_S_* analyses [67]. Furthermore, an ORF (*pvdE*) containing one of the SNPs identified in that earlier analysis was identified in the current study as being under differential selection in CF- and environmental isolates.

In summary, we present here a *d_N_/d_S_* analysis of CF- and environmental *P. aeruginosa* isolates from the IPCD. Our data indicate that whereas most [PAO1] ORFs are strongly conserved, over 200 manifest differential signatures of selection in the two classes of isolate. The overlap between this study and that of previous researchers focusing on commonly encountered pathoadpative mutations *per se* (as opposed to the selection signatures studied here) is relatively small, indicating that a *d_N_/d_S_*-based strategy offers fresh insights into habitat-specific adaptation.

### Significance

The airways of people with CF are often colonized by *P. aeruginosa*. In this work, we show that around 200 genes in the organism manifest signatures of differential selection in CF-associated isolates compared with environmental isolates. Many of these genes have not been previously recognized as important in adaptation to the CF airway environment. Commensurate with predictions from metabolic control theory, we find a statistically significant enrichment of selection at the enzymes controlling metabolic branchpoints. We also identify a region of higher-than-expected gene loss among CF isolates in the Psl polysaccharide biosynthetic gene cluster. However, this gene loss appears to be offset by the acquisition of compensatory mutations in a gene controlling biosynthesis of a less immunogenic polysaccharide, alginate.

## Disclaimer

Copyright permission for the KEGG map pae01100 has been obtained from Kanehisa Laboratories (request reference: 252297).

## Supplementary Material

ugaf040_Supplemental_Files

## Data Availability

All data generated in this study are freely available through the indicated links. Our pipeline is available at https://github.com/ph-u/dNdS_scan and https://doi.org/10.5281/zenodo.17714444. Further details are included in Supplementary Information. Data is publicly available via Zenodo (https://doi.org/10.5281/zenodo.16393514).
