# Supplementary material for: Genome-wide evolutionary selection pressures acting on Pseudomonas aeruginosa residing in different environments
Source: NAR Mol Med. 2025 Nov 29;2(4):ugaf040. doi: 10.1093/narmme/ugaf040 (PMC12771364; doi:10.1093/narmme/ugaf040)
Supplement: ugaf040_Supplemental_Files [file ugaf040_Supplemental_Files.zip › Supplementary Information.pdf]

Supplementary Information

Genome-wide evolutionary selection pressures acting on *Pseudomonas aeruginosa* residing in different environments.

Pok-Man Ho, Rahan Rudland Nazeer, *Martin Welch

Department of Biochemistry, University of Cambridge, Cambridge, United Kingdom

*Corresponding author: mw240@cam.ac.uk

Running title: d_N/d_S analysis of the *P. aeruginosa* genome

Keywords: *Pseudomonas aeruginosa*; cystic fibrosis; d_N/d_S; evolution; computational biology; genomics.

Supplementary Methods

Database preparation. PAO1 genome (both annotated and unannotated) and its annotations [GCF_000006765.1] was downloaded from the *Pseudomonas* Genome DB (1). This reference genome contains 5713 annotated sequences (in the downloaded "CDS" FASTA file), with 5586 ORFs (annotated as "cds"), 63 tRNAs, 45 non-coding RNAs, 12 rRNAs, 6 pseudogenes and 1 transfer-messenger RNA.

Draft genomes with habitat annotations, such as the initial collection of the International *Pseudomonas* Consortium Database (IPCD) [BioProject PRJNA325248] (2), was downloaded from NCBI. This collection has 854 scaffold-level assemblies from isolates collected across the globe, with metadata including sampling country and the sample source for the *Pseudomonas aeruginosa* (PA) isolates. The largest number of isolates were from Canada (283 isolates, 33% of the dataset), Australia (164 isolates, 19%) and the UK (110 isolates, 13%). There were 5 sources of isolates we identified, including cystic fibrosis (CF)-associated (367 isolates [43%], including sputum and throat samples), environmental (116 isolates [14%], including natural/rural settings, various natural water bodies, and food), other infections (265 isolates [31%], including open wounds, burn wounds, blood, urine, eye, acute infections, and non-CF airways), urban (69 isolates [8%], including non-wounded skin, hospital environment, and community facilities) and unknown (37 isolates [4%], unspecified sampling sources). We only reported here the contrast between CF-associated and environmental isolates (483 isolates) as the adaptation of an environmental isolate into a pathogenic lifestyle was one of the concerns for PA among people with Cystic Fibrosis (pwCF). We did not "de-duplicate" isolates from the collection because (1) sequence type (ST) duplication was rare in the IPCD collection we analyzed, (2) most duplicated STs were from different countries, and (3) the metadata did not disclose whether different isolates were obtained from the same host or not. We also note that by using the PAO1 genome as a reference, some strain-specific genes (e.g. in PA14 and LESB58) were not captured in our analyses. However, we note that the PAO1 genome is the best characterized of all *P. aeruginosa* genome sequences, and that its genes

comprise 82-88% of the genome from each of the 483 isolates we analyzed in the IPCD (*data not shown*).

Sequence Type catalogue. Identification of sequence types of all CF-/environmental *P. aeruginosa* isolates was done on an online platform PathogenWatch (v23.2.1) following the widely-adopted multi-locus sequence typing (MLST) approach (3). This revealed the IPCD initial collection had a wide coverage of sequence types (i.e. one isolate per sequence type), with a few exceptions (**Figure 1B**). Yet, all except one of these exceptions were having isolates from both sources. There were in total 101 isolates identified as either ST17, ST27, ST146, ST155, ST179, and ST244. To see whether these multi-isolates sequence types were monophyletic, we performed a whole genome phylogenetic reconstruction using a maximum likelihood approach [PAO1 (GCF_000006765.1/NC_002516.2) as the reference] via the online platform REALPHY.

CDS identification and extraction from the unannotated genome assemblies. Each CDS was matched against our database described earlier reporting sequences mapping to all 854 isolates using *blastn* from BLAST+ (v2.13.0). To eliminate the risk of capturing duplicated or repurposed genes, we compare the flanking regions of 100 nucleotides from both reference and the isolates, before and after the matched sequence output from *blastn*. No flanking region outliers were observed (*data not shown*).

Contrasting habitat-specific contributions to a whole-ORF level d_N/d_S. The d_N/d_S calculation was based on the method implemented by Gömer *et al* in the software vNvS (4)]. The whole-ORF d_N/d_S reported a considerable amount of NAs due to different complications, mainly due to identical sequences and frame-shifting indels. These are the known limitations of the d_N/d_S equation. The range of NAs was mainly between 10-35% depending on the level of conservation of an ORF (*data not shown*), with a couple of exceptions (**Table S1**). Nevertheless, we wanted to test whether habitat-specific signals manifested at the ORF level.

For the first approach, we defined the threshold of “neutral selection / genetic drift” upper boundary as the median of all ORFs that carried a whole-ORF d_N/d_S > 1 and the lower boundary as the reciprocal of the upper boundary. This gave the range of d_N/d_S values as 0.73-1.36, categorized as “neutral selected” ORFs. The lower boundary did not fit our categorization purpose if the median as all ORF with d_N/d_S < 1 was taken as most ORFs in PA were highly conserved (this median is about d_N/d_S = 0.1).

As a second approach, we focused on the numeric contrast between d_N/d_S values of ORFs originated from isolates in the two habitats. We noticed the non-negligible portion of NAs and the disproportionate representation of d_N/d_S on a linear scale. As a compromise, we standardized the contrast by using an equation “[-1 if d_N/d_S of CF < env; 1 if *vice versa*] * log2(absolute value(log10([median of CF-associated isolates] : [median of environmental isolates])))”, which emphasized the absolute scale of differences (using log10) between the available d_N/d_S values between isolates from the two habitats. Here, we used the log2 value of this contrast to comply with the thresholds widely-adopted in proteomics in understanding significant differences of signals. We also noticed that there were no cases of log10 scaled d_N/d_S values between 0-1 (i.e. log2 values would always be positive numbers), hence we took the advantage of the positive/negative signs to segregate between habitats.

Using this d_N/d_S contrast approach, we extracted a list of ORFs manifesting high differences between habitats. To understand possible enrichments of biological functions within this list, an ensemble STRING (v.12.0) analysis was adopted. STRING leveraged a deep learning-based model to annotate links between ORFs. Hence the STRING results were not always reproducible, especially between groups of ORFs with intertwining relationships. To minimize this ambiguity, we deployed the ensemble method by repeated (N=7) STRING analysis of a k-means grouping of 20 clusters. We alternated the k-means clustering of 20 groups and another random grouping number and performed a hierarchical clustering of 20 consensus groups on the 7 replicates of 20 k-means clusters to yield protein functional inferences.

Evaluating whether environments alter the d_N/d_S values between essential and non-essential genes. Turner *et al* (5) proposed a list of essential, conditionally-essential and non-essential genes in the PAO1 genome. Pairwise Wilcox rank sum test with Bonferroni p-value correction was used to test the segregation of the d_N/d_S values of the genes grouped respectively in these three categories.

Evaluating whether branch point metabolites carry signals of habitat-specific selection pressure. We also collected a list of metabolites linked with multiple metabolic reactions showing habitat-specific differences. We deployed Wilcox rank sum test to test whether there was a significant enrichment of differential selection on either CF-associated or environmental isolates.

Contrasting habitat-specific contributions to a residue-level ORF d_N/d_S. Residue-level ORF d_N/d_S reconstruction was calculated as the mean value of all d_N/d_S values of sliding windows that included the residue of interest. To contrast the sampling environment contribution on the evolution selectivity between CF and environmental isolates, we deployed the Wilcox rank sum test with Benjamini-Hochberg false discovery rate p-value correction.

Sliding window is chosen as the largest window size out of three options: 67, 33 and 7 codons. (The size of window does not affect the result but a small window on a long sequence would generate an unnecessarily large amount of data that do not add much value onto downstream analyses.) Each sliding window started from the first residue and ended at the last without overshooting the sequence or shrinking the window size at both ends of an ORF. If an inframe insertion/deletion (indels) was detected between PAO1 and the IPCD-NCBI sequences, the gene segments before and after an indel were treated as independent fragments. Identical sequence windows were set as $d_N/d_S = 0$. This set the foundation for a statistically-robust residue-level reconstruction, as we assumed an extreme form of residue conservation was the conservation of nucleotide sequences. Residue-level reconstruction was taken as the central tendency measures (i.e. minimum, first quartile, second quartile/median, third quartile, maximum, and mean) from all sliding windows containing the residue-of-consideration.

Contrasting *mucA* conservation between CF-associated isolates with intact or non-intact *psl* operon. Nucleotide sequences of PA0763 (*mucA*) from CF-associated isolates were segregated into two groups. Grouping was according to whether the *psl* operon in the respective isolate was intact [all PA2231-45 (*pslA-O*) genes were identified by *blastn*] or not. Among the 310 CF-associated isolates with intact *psl* operons and 57 without, we performed a multi-sequence alignment and calculated the proportion of nucleotide usage at every position for the respective group. Then we contrasted the nucleotide usage between the two

groups at every nucleotide position. Observable enrichments were extracted and we employed an *in silico* mutation approach of the PAO1 *mucA* gene. Comparing this *mucA* variant (consensus among clinical isolates) to the PAO1 reference yielded corresponding synonymous, missense and nonsense mutations.

Computational tools. The d_N/d_S pipeline was carried out on the Cambridge High Performance Computing Cluster (CSD3 Icelake-himem cluster) with R (v4.3.1) and R packages "ape" (v5.8) and "Biostrings" (v2.68.1). Statistical tests were performed locally with R (v4.1.2). Graphics were constructed with various R packages, including "lattice" (v0.22.6), "ggplot2" (v3.5.1), "ggmap" (v4.0.0), "scatterpie" (v0.2.3), "ggrepel" (v0.9.5), "EnhancedVolcano" (v1.12.0), "msa" (v1.26.0), "WeightedTreemaps" (0.1.4), "rlang" (v1.1.4), "stringr" (v1.5.1) and "gridExtra" (v2.3). Protein structures were visualized in PyMol Open-Source (v2.5.0) and python3 (v3.10.12).

Supplementary Figures

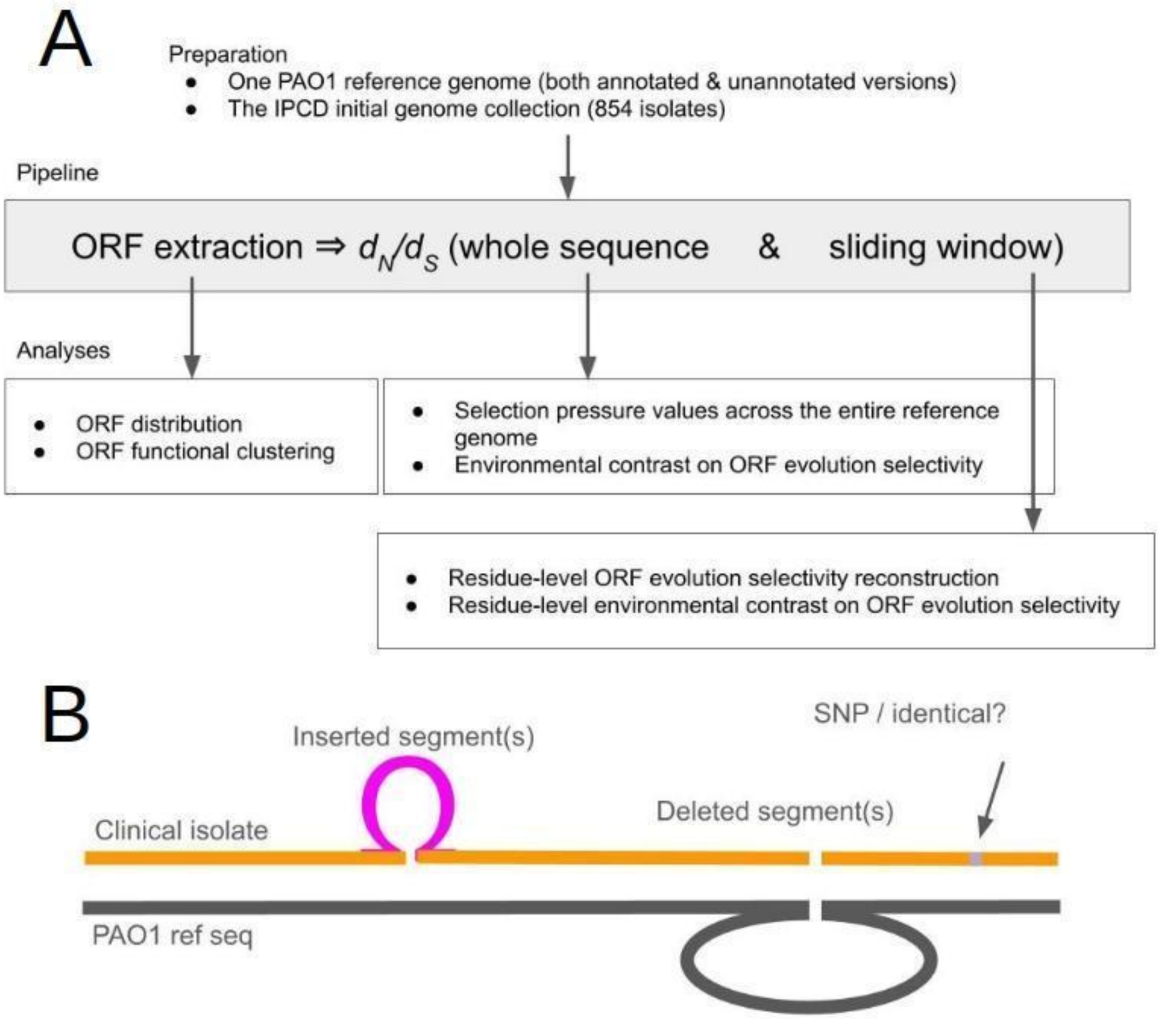

Figure S1: Overview of the analysis workflow. (**A**) Our pipeline consisted of two stages: the ORF extraction stage and d_N/d_S calculations with two sizes of sequence window. This approach enables a multi-level evaluation of an ORF, including its distribution and sequence integrity within a population, its distribution in relation to other ORFs, and the environmental contributions on the evolution selectivity regarding both the whole ORF and residue levels. (**B**) Illustration showing how we augment the d_N/d_S calculation to also capture ORFs with indels that do not disrupt the reading frame. If in-frame indels were detected in either the reference or sample genome, the respective extra codon segments were identified and excluded through sequence alignment before the d_N/d_S calculation step.

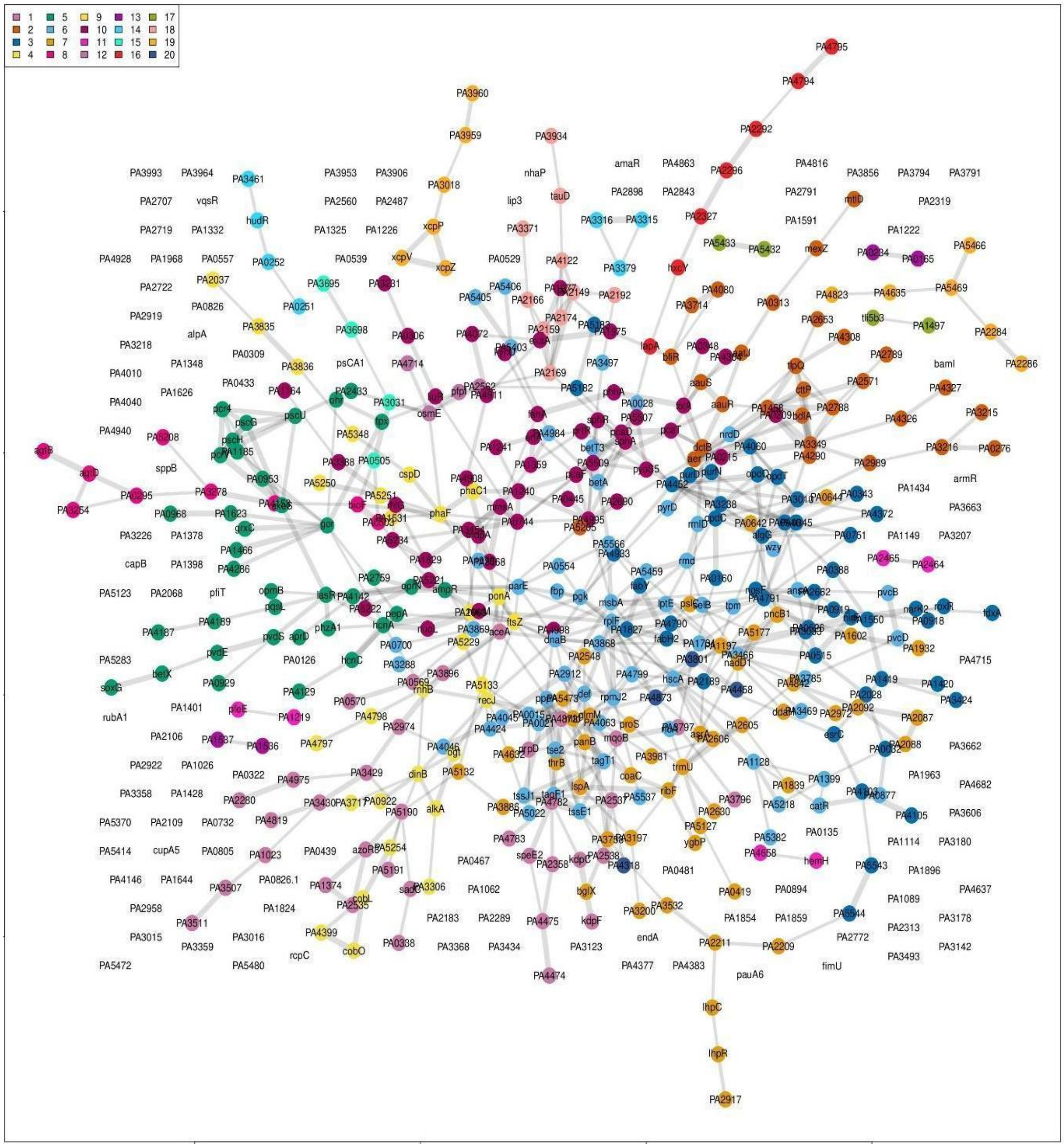

Figure S2: Consensus STRING network showing functional clusters of ORFs associated with differential selection in CF and environmental isolates. ORFs represented by the 530 red data points in the Volcano plot (**Figure 2B**) were subjected to iterative *k*-means clustering as outlined in the body text to yield 20 statistically robust functional clusters (indicated by different colours in the figure).

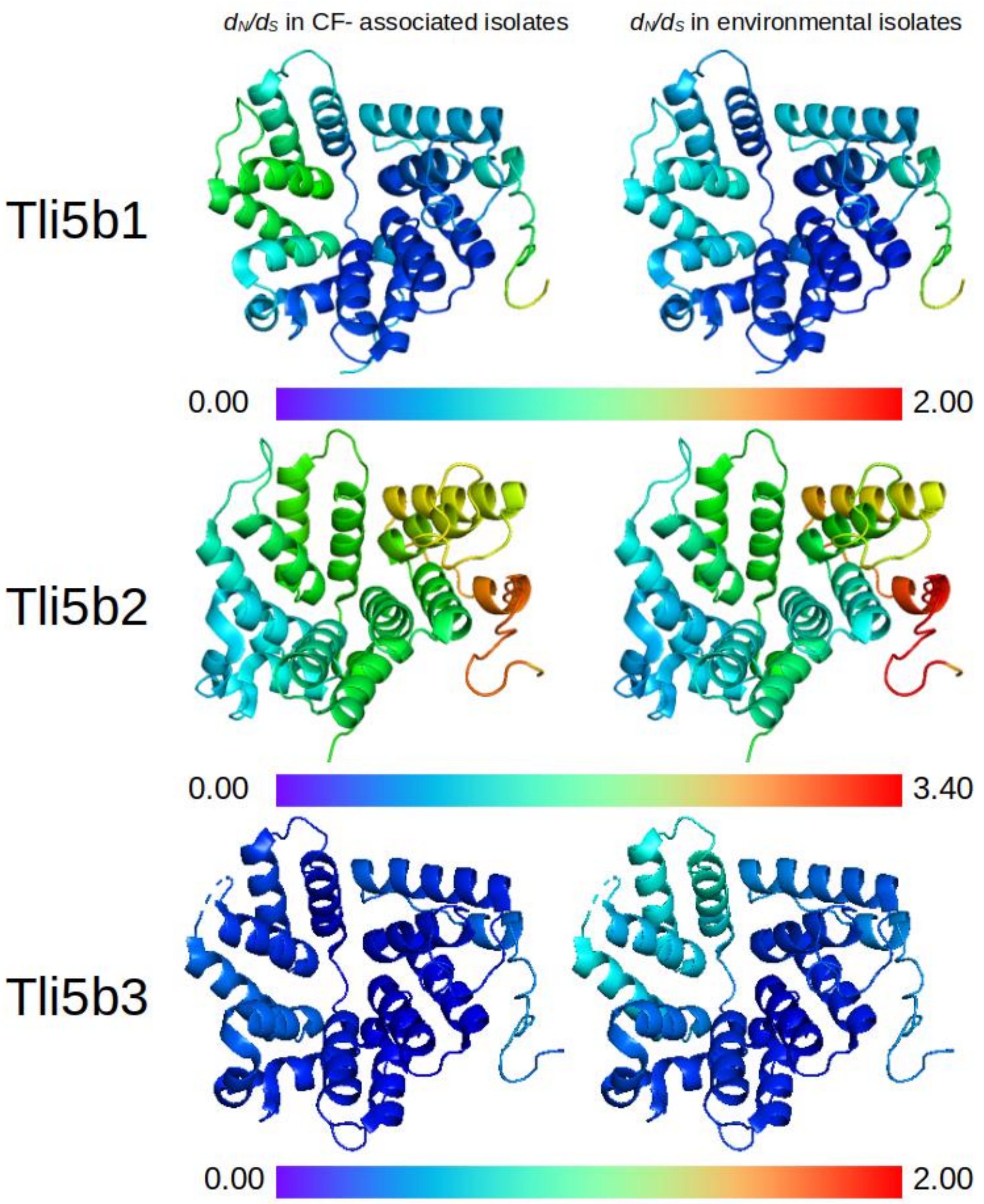

Figure S3: Residue-by-residue d_N/d_S analysis of the Type VI Secretion lipase immunity proteins, Tli5b1, Tli5b2, and Tli5b3. The figure shows the codon-by-codon d_N/d_S output for Tli5b1, Tli5b2, and Tli5b3 mapped onto the X-ray crystal structures of the proteins (PDB codes 6LCH, 5JKP, and 5JJO, respectively). Note that although the median d_N/d_S value for each ORF was similar (median *dN/dS* values for *tli5b1* were 0.091 (CF) and 0.100 (Env); median *dN/dS* values for *tli5b2* were 0.131 (CF) and 0.131 (Env); median *dN/dS* values for *tli5b3* were 0.093 (CF) and 0.110 (Env)) the fine-grained pattern of d_N/d_S across each protein was variable, with some parts of the structures such as the low complexity region at the N-terminus (6) manifesting different signatures of selection in the CF- and environmental strains.

Supplementary Tables

Table S1. The Table shows the numbers of ORFs displaying signatures of negative selection, neutral drift, or positive selection in the CF-associated and environmental IPCD isolates. For example, 5178 ORFs were found to be negatively selected in both the CF-associated and environmental isolates, whereas 34 ORFs were found to be undergoing drift (neutral selection) in the CF-associated isolates but were negatively selected in the environmental isolates. Numbers shaded in gray highlight counts of ORFs displaying the indicated differential selection signatures. In a small number of cases, d_N/d_S was not calculable, as indicated. The reasons for this were *(i)* most/all clinical isolates did not encode the corresponding ORF (PA0715, PA0716, PA1369, PA1371, PA1936, PA1939, PA2730, PA2731, PA2733, PA2734, PA2735, PA3156 (*wbpD*)), *(ii)* the ORF was completely conserved (PA0826.1, PA4255 (*rpmC*), PA5276 (*lppL*), PA5471.1) in all isolates or *(iii)* the reference (PAO1) sequence contained at least one frameshift (PA1029, PA1122, PA1134, PA1174 (*napA*), PA1327, PA1559, PA1685 (*masA*), PA1729, PA2139, PA2141, PA2492 (*mexT*), PA2547, PA2727, PA2928, PA4059, PA4360, PA5412, PA5525, PA5532).

		Selective pressure in environmental isolates			
		Negative	Neutral	Positive	d_N/d_S not calculable
Selection pressure in CF-isolates	Negative	5178	12	27	8
	Neutral	34	41	5	0
	Positive	109	19	126	1
	d_N/d_S not calculable	3	0	2	21

Table S2 [Supplementary Dataset]. The table shows a list of the ORFs displaying non-negative selection signatures, along with their respective d_N/d_S values. Blocks of ORFs are colour coded to indicate their respective categories in **Table S1** e.g., the 12 ORFs that are subject to drift in the environmental isolates but negative selection in the CF-associated isolates are coloured grey, whereas the 27 ORFs shaded in green are undergoing negative selection in the CF isolates but positive selection in the environmental isolates, and so on.

Table S3: List of ORFs displaying quantifiable signatures of differential selection in isolates from pwCF and isolates from the environment. Out of the 530 ORFs highlighted in red in **Figure 2B**, 99 ORFs displayed variable d_N/d_S values among isolates from pwCF but were highly conserved in environmental isolates. By contrast 15 ORFs displayed variable d_N/d_S values among environmental isolates but were highly conserved in isolates from pwCF. The lists are ordered in descending -$\log_{10}$ *p* value. The locus ID, -$\log_{10}$ [*p* value], and if available, the gene/ORF name, are shown. ORFs previously demonstrated to be essential *in vitro* are highlighted in bold (see Turner *et al* (2015)).

(i) ORFs displaying variability in CF-associated isolates but conservation in environmental isolates.				
PA4729 (15.7, *panB*)	PA2087 (15.7)	PA3959 (11.9)	PA1420 (9.6)	PA0102 (9.6, *pscA1*)
PA3642 (8.9, *rnhB*)	PA1839 (8.3)	PA1114 (7.9)	PA0968 (7.9)	PA2255 (7.7, *pvcB*)
PA2538 (7.5)	PA4196 (6.9, *bfiR*)	PA3794 (6.9)	**PA4931 (6.8, *dnaB*)**	PA1968 (6.8)
PA5406 (6.7)	PA4795 (6.7)	PA2059 (6.6, *sppB*)	PA0080 (6.2, *tssJ1*)	PA4046 (5.7)
PA5191 (5.6)	PA5129 (5.6, *grxC*)	PA4424 (5.5)	PA1635 (5.2, *kdpC*)	PA5566 (5.1)
PA0338 (5.1)	PA4908 (5.0)	PA1164 (5.0)	PA5229 (4.9)	PA3278 (4.9)
PA0526 (4.9)	PA4364 (4.8)	PA2257 (4.8, *pvcD*)	**PA2169 (4.7)**	PA2707 (4.6)
PA1430 (4.6, *lasR*)	PA3178 (4.5)	PA5495 (4.4, *thrB*)	PA5127 (4.4)	PA3454 (4.4)
PA1623 (4.4)	PA0872 (4.2, *phhA*)	PA2974 (4.1)	PA2789 (4.0)	PA2364 (4, *lip3*)
PA0231 (4, *pcaD*)	PA0225 (3.9, *erfA*)	PA2016 (3.8, *liuR*)	PA1226 (3.8)	PA0160 (3.8)
PA3791 (3.6)	PA3226 (3.5,)	PA0481 (3.5)	PA5480 (3.4)	PA4799 (3.4)
PA4782 (3.4)	PA1963 (3.3)	PA1721 (3.3, *pscH*)	PA5403 (3.2)	PA2958 (3.2)
PA4040 (3.1)	PA2286 (3.1,)	PA1702 (3.1, *pcr4*)	PA4674.1 (3, *higB*)	PA3856 (3.0)
PA1359 (3.0)	PA1219 (3.0)	PA0215 (2.8)	PA3621 (2.7, *fdxA*)	**PA4248 (2.6, *rplF*)**
PA2634 (2.6, *aceA*)	PA2898 (2.5)	PA0467 (2.5)	PA3033 (2.4)	PA0918 (2.4)
PA5351 (2.3, *rubA1*)	PA1026 (2.3)	PA2832 (2.1, *tpm*)	PA0505 (2.1)	PA5348 (2.0)
PA3207 (2.0)	PA3123 (2.0)	PA2989 (2.0)	PA1240 (2.0)	**PA0805 (2.0)**
PA4876 (1.9, *osmE*)	PA3316 (1.9)	PA2313 (1.9)	PA0251 (1.9)	PA3663 (1.8)
PA3511 (1.8)	PA3016 (1.8)	PA1720 (1.8, *pscG*)	PA3104 (1.7, *xcpP*)	PA2280 (1.7)
PA0985 (1.7, *pyoS5*)	PA3698 (1.6)	PA2464 (1.6)	PA2622 (1.5, *cspD*)	
(ii) ORFs displaying variability in environmental isolates but conservation in CF-associated isolates.				
PA3266 (9.4, *capB*)	PA1325 (6.1)	PA0922 (3.8)	PA0135 (3.5)	PA4105 (3.4)
PA5060 (2.8, *phaF*)	PA2106 (2.5)	PA2683.1 (2.2, *tsi5*)	PA2159 (2.2)	PA1632 (2, *kdpF*)
PA4940 (1.7)	PA0729 (1.7, *pfiT*)	PA4377 (1.5)	PA2772 (1.5)	PA3865.1 (1.4)

Table S4: Ensemble STRING analysis on the differential ORF evolutionary conservation between CF and environmental isolates. Out of the 530 ORFs highlighted red in **Figure 2B**, 408 were assigned to networks. Replicates (N = 7) of 20 groups k-means clustering was performed on STRING (v12.0) (network IDs: bEWBSxQpQFFZ, biR0iZbvIxTw, bpd3VGtqqoZV, b5at7w7yfOeB, bWAKjpkTHMsJ, bsOb1aatkEhE, bvclVZO1aWtJ). A hierarchical clustering was performed in R (v4.1.2) on the clustering output from each of the 7 replicates and 20 k-means groups were resegregated. Bolded texts overlap with the proposed list of high mutational burden genes (Weimann et al., 2024). Functional enrichment networks for each cluster are accessible via the permalink https://version-12-0.string-db.org/cgi/network?networkId=NETWORKID

Cluster number<br>• Number of members<br>• Clustering coefficient (p-value)<br>• Functional enrichment<br>• STRING network ID	Genes significantly more conserved in … than the other	
	CF isolates	Environmental isolates
1<br>• 35<br>• 0.46 (<<0.01)<br>• No significant<br>• b8redntqcJEA	PA1632 [*kdpF*]<br>PA2358<br>PA2535<br>PA2537<br>PA3223 [*azoR3*]<br>PA3429<br>PA3430<br>PA3796<br>PA3797<br>PA3896<br>PA4332 [*sadC*]<br>PA4474<br>PA4475<br>PA4640 [*mqoB*]<br>PA4774 [*speE2*]<br>PA4819<br>PA4975<br>PA5190	PA0338<br>PA0570<br>PA0792 [*prpD*]<br>PA1023<br>PA1374<br>PA1635 [*kdpC*]<br>PA2280<br>PA2538<br>PA2634 [*aceA*]<br>PA2974<br>PA3507<br>PA3511<br>PA4782<br>PA4783<br>PA4872<br>PA5191
2<br>• 29<br>• 0.625 (<<0.01)<br>• Chemotaxis, signal transduction<br>• by2idegKILV0	PA0180 [*cttP*]<br>PA0276<br>PA1336<br>PA1342 [*aatJ*]<br>PA1423 [*bdlA*]<br>PA1458<br>PA1561 [*aer*]<br>PA2342 [*mtlD*]<br>PA2571<br>PA2653<br>PA2654<br>PA2788<br>PA3215<br>PA3216	PA0313<br>PA1335<br>**PA2020 [*mexZ*]**<br>PA2789<br>PA2989<br>PA4196 [*bfiR*]<br>PA4290

	PA3349<br>PA3714<br>PA4080<br>PA4308<br>PA4326<br>PA4327<br>PA5165 [*dctB*]<br>PA5205	
3<br>• 48<br>• 0.549 (<<0.01)<br>• Mixed metabolism, nitrogen metabolism<br>• b5ut0kxybQCb	PA0343<br>PA0388<br>PA0751<br>PA0919<br>PA0946<br>PA1550<br>PA1827<br>PA2028<br>PA2189<br>PA2505 [*opdT*]<br>PA2662<br>PA3010<br>PA3038 [*opdQ*]<br>PA3238<br>PA3333 [*fabH2*]<br>PA3394 [*nosF*]<br>PA3424<br>PA3876 [*narK2*]<br>PA4060<br>PA4105<br>PA4372<br>PA4493 [*roxR*]<br>PA4596 [*esrC*]<br>PA4791<br>PA5182<br>PA5183<br>PA5543<br>PA5544	PA0032<br>PA0160<br>PA0162 [*opdC*]<br>PA0345<br>PA0515 [*nirD2*]<br>PA0516 [*nirF*]<br>PA0526<br>PA0918<br>PA0944 [*purN*]<br>PA1419<br>PA1420<br>PA3033<br>**PA3545 [*algG*]**<br>PA3621 [*fdxA*]<br>PA4103<br>PA4452<br>PA4790 [*cmoM*]<br>PA4855 [*purD*]<br>PA4933<br>PA5174 [*fabY*]
4<br>• 14<br>• 0.605 (<<0.01)<br>• DNA interaction<br>• bYW0tmevgmuf	PA0922<br>PA0923 [*dinB*]<br>PA2907 [*cobL*]<br>PA3306<br>PA3725 [*recJ*]<br>PA5254 [*fkl*]	PA0995 [*ogt*]<br>PA1272 [*cobO*]<br>PA1686 [*alkA*]<br>PA3642 [*rnhB*]<br>PA3717<br>PA4399<br>PA4797<br>PA4798
5<br>• 37<br>• 0.674 (<<0.01)<br>• T3SS, virulence, glutathione metabolism<br>• bsOV8zfM596D	PA0929<br>PA1185<br>PA1246 [*aprD*]<br>PA1690 [*pscU*]<br>PA1706 [*pcrV*]<br>PA2025 [*gor*]<br>PA2193 [*hcnA*]<br>PA2195 [*hcnC*]<br>PA2495 [*oprN*]<br>PA2525 [*opmB*]	PA0953<br>PA0968<br>**PA1430 [*lasR*]**<br>PA1466<br>PA1623<br>PA1702 [*pcr4*]<br>PA1720 [*pscG*]<br>PA1721 [*pscH*]<br>PA2397 [*pvdE*]<br>**PA2426 [*pvdS*]**

	PA2532 [*tpx*]<br>PA2850 [*ohr*]<br>PA3831 [*pepA*]<br>PA3841 [*exoS*]<br>PA4109 [*ampR*]<br>PA4187<br>PA4189<br>PA4190 [*pqsL*]	PA2433<br>PA2759<br>PA3236 [*betX*]<br>PA4129<br>PA4142<br>PA4210 [*phzA1*]<br>PA4286<br>PA5129 [*grx*]<br>PA5419 [*soxG*]
6<br>● 63<br>● 0.536 (<<0.01)<br>● Mixed metabolism, T6SS<br>● bFm4jECGz67G	PA0015<br>PA0019 [*def*]<br>PA0073 [*tagT1*]<br>PA0075 [*pppA*]<br>PA0076 [*tagF1*]<br>PA0087 [*tssE1*]<br>PA0554<br>PA0877<br>PA1734<br>PA1920 [*nrdD*]<br>PA2253 [*ansA*]<br>PA2912<br>PA3050 [*pyrD*]<br>PA3246 [*rluA*]<br>PA3288<br>PA3785<br>PA3810 [*hscA*]<br>PA3868<br>PA3869<br>PA3988 [*lptE*]<br>PA4045<br>PA4063<br>PA4807 [*selB*]<br>PA4967 [*parE*]<br>PA4970<br>PA4984<br>PA4997 [*msbA*]<br>PA5022<br>PA5110 [*fbp*]<br>PA5218<br>PA5372 [*betA*]<br>PA5405<br>PA5454 [*rmd*]<br>PA5459	PA0021<br>PA0028<br>PA0080 [*tssJ1*]<br>PA0552 [*pgk*]<br>PA0700<br>PA1128<br>PA1399<br>PA2255 [*pvcB*]<br>PA2257 [*pvcD*]<br>PA2510 [*catR*]<br>PA2702 [*tse2*]<br>PA2832 [*tpm*]<br>PA3154 [*wzy*]<br>PA3466<br>PA3469<br>PA3497<br>PA3600 [*rpmJ2*]<br>PA3933 [*betT3*]<br>PA4046<br>PA4248 [*rplF*]<br>PA4424<br>PA4799<br>PA4931 [*dnaB*]<br>PA5162 [*rmlD*]<br>PA5382<br>PA5403<br>PA5406<br>PA5537<br>PA5566
7<br>● 48<br>● 0.463 (<<0.01)<br>● Mixed metabolisms<br>● b4pCZbp2btKm	PA0419<br>PA0642<br>PA0779 [*lon2*]<br>PA0956 [*proS*]<br>PA1197 [*cobB2*]<br>PA1254 [*lhpC*]<br>PA1602<br>PA1726 [*bglX*]<br>PA1932<br>PA2088<br>PA2209	PA0644<br>PA1195 [*ddaH*]<br>PA1261 [*lhpR*]<br>PA1839<br>PA2087<br>PA2092<br>PA2233 [*pslC*]<br>PA2548<br>PA2605 [*tusD*]<br>PA2606 [*tusC*]<br>PA2626 [*trmU*]

	PA2211<br>PA2630<br>PA3002 [*mfd*]<br>PA3197<br>PA3758<br>PA3981<br>PA4006 [*nadD*]<br>PA4559 [*lspA*]<br>PA4561 [*ribF*]<br>PA4632<br>PA4842<br>PA4919 [*pncB1*]<br>PA5177<br>PA5320 [*coaC*]<br>PA5473	PA2917<br>PA2972<br>PA3200<br>PA3532<br>PA3633 [*ygbP*]<br>PA3886<br>PA4729<br>PA4749 [*glmM*]<br>PA5127<br>PA5132<br>PA5495 [*thrB*]
8<br>● 9<br>● 0.519 (<<0.01)<br>● Biotin biosynthesis, quorum sensing<br>● bSaLQWm3XEoB	PA0295<br>PA0503 [*bioC*]<br>PA0604 [*agtB*]<br>PA0606 [*agtD*]<br>PA4153	PA0501 [*bioF*]<br>PA3254<br>PA3278<br>PA5208
9<br>● 15<br>● 0.444 (<<0.01)<br>● Polymer metabolisms<br>● bFPcUTnG5Shd	PA1531<br>PA2037<br>PA2963 [*mltG*]<br>PA3835<br>PA3836<br>PA5045 [*ponA*]<br>PA5060 [*phaF*]<br>PA5250	PA2622 [*cspD*]<br>PA4407 [*ftsZ*]<br>PA5056 [*phaC1*]<br>PA5133<br>PA5229<br>PA5251<br>PA5348 [*hupA*]
10<br>● 48<br>● 0.543 (<<0.01)<br>● Catabolisms<br>● bBJEMVgzykCQ	PA0228 [*pcaF*]<br>PA0229 [*pcaT*]<br>PA0348<br>PA0611 [*prtR*]<br>PA0744<br>PA1241<br>PA1829<br>PA1975<br>PA1982 [*exaA*]<br>PA2008 [*fahA*]<br>PA2080 [*kynU*]<br>PA2647 [*nuoL*]<br>PA2648 [*nuoM*]<br>PA3231<br>PA3388 [*rcsF*]<br>PA3568<br>PA3570 [*mmsA*]<br>PA4498 [*mdpA*]<br>PA4995<br>PA4998<br>PA5234<br>PA5324 [*sphR*]<br>PA5325 [*sphA*]<br>PA5507<br>PA5509	PA0209 [*mdcB*]<br>PA0215<br>PA0225 [*erfA*]<br>PA0231 [*pcaD*]<br>PA0306<br>PA0350 [*folA*]<br>PA0445<br>PA0872 [*phhA*]<br>PA0985 [*pyoS5*]<br>PA1164<br>PA1240<br>PA1359<br>PA1977<br>PA2016 [*liuR*]<br>PA2690<br>PA3387 [*rhlG*]<br>PA3454<br>PA4072<br>PA4364<br>PA4908<br>PA4911<br>PA5221<br>PA5222

11<br>● 6<br>● 1 (<<0.01)<br>● No significant<br>● bKWlUjXuxrNg	PA2465<br>PA2689 [*pfeE*]<br>PA4655 [*hemH*]<br>PA4658	PA1219<br>PA2464
12<br>● 4<br>● 0.75 (<<0.01)<br>● Mixed uncharacterized<br>● bfIxfilD4uhR	PA0355 [*pfpI*]<br>PA2562	PA4714<br>PA4876 [*osmE*]
13<br>● 4<br>● 1 (<<0.01)<br>● Nucleoside-specific channel-forming protein<br>● bCxEjp3c0t23	PA0165<br>PA1536<br>PA1537	PA0234
14<br>● 7<br>● 0.714 (<<0.01)<br>● Mixed metabolisms<br>● bM6seZQnmbRZ	PA0252<br>PA3315<br>PA3379	PA0251<br>PA0253 [*hudR*]<br>PA3316<br>PA3461
15<br>● 4<br>● 0.5 (<<0.01)<br>● Mixed<br>● bHwchXYb6Eub	PA3031<br>PA3695	PA0505<br>PA3698
16<br>● 7<br>● 0.286 (<<0.01)<br>● Mixed metabolisms<br>● bikdb1MZ5d6W	PA0683 [*hxcY*]<br>PA0688 [*lapA* / *phoA2*]<br>PA2292<br>PA2296<br>PA2327<br>PA4794	PA4795
17<br>● 4<br>● 1 (<<0.01)<br>● Acetyltransferase, sulphur transport<br>● b8udAiY3KAxZ	PA5088 [*tli5b3*]<br>PA5432	PA1497<br>PA5433
18<br>● 10<br>● 0.64 (<<0.01)<br>● Hydrocarbon metabolisms<br>● bLVlok5cAQBT	PA2149<br>PA2159<br>PA2166<br>PA2174<br>PA3371<br>PA3934<br>PA3935 [*tauD*]	PA2169<br>PA2192<br>PA4122
19<br>● 12<br>● 0.528 (<<0.01)<br>● T2SS<br>● bjzj2a4tovlD	PA2284<br>PA3099 [*xcpV*]<br>PA4823<br>PA5466<br>PA5469	PA2286<br>PA3018<br>PA3095 [*xcpZ*]<br>PA3104 [*xcpP*]<br>PA3959<br>PA3960<br>PA4635

20<br>• 4<br>• 0.5 (0.09)<br>• No significant<br>• bz0TGmpWp3sd	PA3801<br>PA4318<br>PA4458<br>PA4873	<NA>

Table S5. Number of ORFs in the indicated KEGG pathways that manifest habitat-specific selection pressures. “Within pathway” ORFs are those that encode enzymes catalyzing the main sequence reactions of a pathway. “Associated” ORFs are those that encode enzymes which act on substrates/products shared by more than one KEGG pathway.

		CF-conserved ORFs		Environmentally-conserved ORFs	
KEGG id	Pathway	Within pathway	Associated	Within pathway	Associated
M00001	Glycolysis (Embden-Meyerhof pathway)	1	-	1	1
M00003	Gluconeogenesis	1	-	1	-
M00307	Pyruvate oxidation	-	-	-	2
M00009	Citrate cycle (TCA cycle, Krebs cycle)	1	4	-	4
M00012	Glyoxylate cycle	1	1	1	1
M00982	Methylcitrate cycle	1	3	2	2
M00171	C4-dicarboxylic acid cycle, NAD - malic enzyme type	1	1	-	1
M00144	NADH:quinone oxidoreductase, prokaryotes	2	-	-	-
M00148	Succinate dehydrogenase (ubiquinone)	-	3	-	1
M00150	Fumarate reductase, prokaryotes	-	3	-	1
M00087	Beta-Oxidation (Fatty acid)	-	1	13	-
M00048	*De novo* purine biosynthesis	-	3	1	-
M00049	Adenine ribonucleotide biosynthesis	-	2	-	-
M00050	Guanine ribonucleotide biosynthesis	-	3	-	-
M00053	Deoxyribonucleotide biosynthesis	-	4	-	-
M00958	Adenine ribonucleotide degradation	3	-	-	-
M00959	Guanine ribonucleotide degradation	2	1	-	-
M00546	Purine degradation	1	1	-	-
M00052	Pyrimidine ribonucleotide biosynthesis	-	2	-	-
M00018	Threonine biosynthesis	-	1	1	-
M00621	Glycine cleavage system	-	2	-	1
M00975	Betaine degradation, bacteria	-	2	1	-
M00034	Methionine salvage pathway	2	-	-	-
M00019	Valine/isoleucine biosynthesis	-	1	-	2
M00535	Isoleucine biosynthesis	-	1	-	1
M00844	Arginine biosynthesis	-	3	-	-
M00023	Tryptophan biosynthesis	-	3	-	-
M00044	Tyrosine degradation	2	-	-	1
M00533	Homoprotocatechuate degradation	-	-	2	-
M00118	Glutathione biosynthesis	-	5	-	2
M00948	Hydroxyproline degradation	1	3	-	-
M00909	UDP-GlcNAc biosynthesis, prokaryotes	-	2	-	-
M00895	Thiamine biosynthesis, prokaryotes	-	2	-	1
M00125	Riboflavin biosynthesis, plants and bacteria	2	1	-	-
M00115	NAD biosynthesis	1	4	-	-
M00120	Coenzyme A biosynthesis	2	-	-	-
M00572	Pimeloyl-ACP biosynthesis, BioC-BioH pathway	1	-	-	1
M00123	Biotin biosynthesis	-	-	2	-
M00126	Tetrahydrofolate biosynthesis	-	1	1	3
M00140	C1-unit interconversion, prokaryotes	-	-	1	3
M00924	Cobalamin biosynthesis, anaerobic	2	-	-	1
M00925	Cobalamin biosynthesis, aerobic	1	-	-	1
M00122	Cobalamin biosynthesis	-	-	1	1
M00835	Pyocyanine biosynthesis	-	-	2	-

M00637	Anthranilate degradation	-	2	-	-

References

1. Winsor,G.L., Griffiths,E.J., Lo,R., Dhillon,B.K., Shay,J.A. and Brinkman,F.S.L. (2016) Enhanced annotations and features for comparing thousands of Pseudomonas genomes in the Pseudomonas genome database. *Nucleic Acids Res.*, **44**, D646-653.

2. Freschi,L., Vincent,A.T., Jeukens,J., Emond-Rheault,J.-G., Kukavica-Ibrulj,I., Dupont,M.-J., Charette,S.J., Boyle,B. and Levesque,R.C. (2019) The *Pseudomonas aeruginosa* Pan-Genome Provides New Insights on Its Population Structure, Horizontal Gene Transfer, and Pathogenicity. *Genome Biol. Evol.*, **11**, 109–120.

3. Curran,B., Jonas,D., Grundmann,H., Pitt,T. and Dowson,C.G. (2004) Development of a Multilocus Sequence Typing Scheme for the Opportunistic Pathogen *Pseudomonas aeruginosa*. *J. Clin. Microbiol.*, **42**, 5644–5649.

4. Gömer,A., Brown,R.J.P., Pfaender,S., Deterding,K., Reuter,G., Orton,R., Seitz,S., Bock,C.-T., Cavalleri,J.M.V., Pietschmann,T., *et al.* (2022) Intra-host analysis of hepaciviral glycoprotein evolution reveals signatures associated with viral persistence and clearance. *Virus Evol.*, **8**, veac007.

5. Turner,K.H., Wessel,A.K., Palmer,G.C., Murray,J.L. and Whiteley,M. (2015) Essential genome of *Pseudomonas aeruginosa* in cystic fibrosis sputum. *Proc. Natl. Acad. Sci.*, **112**, 4110–4115.

6. Mier,P. and Andrade-Navarro,M.A. (2021) The Conservation of Low Complexity Regions in Bacterial Proteins Depends on the Pathogenicity of the Strain and Subcellular Location of the Protein. *Genes*, **12**, 451.
